# Peptide-oligonucleotide conjugates exhibiting pyrimidine-X cleavage specificity efficiently silence miRNA target acting synergistically with RNase H

**DOI:** 10.1038/s41598-018-33331-z

**Published:** 2018-10-09

**Authors:** O. A. Patutina, M. A. Bazhenov, S. K. Miroshnichenko, N. L. Mironova, D. V. Pyshnyi, V. V. Vlassov, M. A. Zenkova

**Affiliations:** Institute of Chemical Biology and Fundamental Medicine SB RAS, Lavrentiev ave., 8, Novosibirsk, 630090 Russia

## Abstract

Taking into account the important role of miRNA in carcinogenesis, oncogenic miRNAs are attractive molecules for gene-targeted therapy. Here, we developed a novel series of peptide-oligonucleotide conjugates exhibiting ribonuclease activity targeted to highly oncogenic miRNAs miR-21 and miR-17. When designing the conjugates, we enhanced both nuclease resistance of the targeted oligodeoxyribonucleotide by introducing at its 3′-end mini-hairpin structure displaying high thermostability and robustness against nuclease digestion and the efficiency of its functioning by attachment of the catalytic construction (amide)NH_2_-Gly(ArgLeu)_4_-TCAA displaying ribonuclease activity to its 5′-end. Designed miRNases efficiently cleaved miRNA targets, exhibiting Pyr-X specificity, and cleavage specificity had strong dependence on the miRNA sequence in the site of peptide location. *In vitro*, designed miRNases do not prevent cleavage of miRNA bound with the conjugate by RNase H, and more than an 11-fold enhancement of miRNA cleavage by the conjugate is observed in the presence of RNase H. In murine melanoma cells, miRNase silences *mmu*-miR-17 with very high efficiency as a result of miR-17 cleavage by miRNase and by recruited RNase H. Thus, miRNases provide a system of double attack of the miRNA molecules, significantly increasing the efficiency of miRNA downregulation in the cells in comparison with antisense oligonucleotide.

## Introduction

Tumor development is accompanied by rapid proliferation, loss of differentiation, development of tumor cell resistance to death, reprogramming of energy metabolism, loss of adhesion between cells and tumor matrix, evasion of immune surveillance, immunosuppression, induction of angiogenesis, infiltration growth, and metastasis^1^. With the discovery of the unique role of non-coding RNAs, in particular microRNAs (miRNAs, miRs), in the regulation of fundamental physiological processes including all stages of tumor progression, interest to use these molecules as potential targets for gene-targeted therapy has arisen^2^.

MicroRNAs are regulatory small noncoding RNAs, with their roles already confirmed to be important for post-transcriptional regulation of gene expression. The functioning of miRNAs is mostly initiated through their binding to the 3′-untranslated region (3′UTR) of the target mRNAs, and results in decreasing target stability and translation efficiency^3–5^. Intracellular and extracellular miRNAs participate in autocrine and paracrine regulation of the expression of their proto-oncogenic and oncosuppressor mRNAs, resulting in either a malignant transformation of the cells or maintenance of its normal homeostasis^6,7^. The use of miRNAs as targets for gene-targeted therapeutics to overcome tumor progression, has led to a paradigm shift that was, until recently, entirely focused on down-regulation of coding mRNAs.

Several strategies have been developed in recent years to inhibit oncogenic miRNAs. Among them is a direct approach that targets mature oncogenic miRNA with an antisense oligonucleotides, antimiRs and miRNA sponges that resulted in the decrease of oncogenic miRNA level in tumor cell and restore normal cell proliferation and sensitivity to apoptotic signals^8,9^. One of the novel directions in inhibition of oncogenic miRNAs is application of artificial ribonucleases which are conjugates of oligonucleotides complementary targeted to miRNAs and catalytic constructions^10–12^. Previously this strategy was used for down-regulation of different viral RNAs^13,14^ and eukaryotic mRNAs^15^ and recently taking into account widespread of miRNA was applied for down-regulation of oncogenic miRNAs^10,11^.

This direction is only beginning to develop. There is one example miRNA-targeted metal-dependent ribonuclease — conjugates of peptide nucleic acid (PNA)-PEG-PNA-PEG with HGG-Cu or DETA, targeted to the *hsa*-miR-1323 which demonstrated effective cleavage of RNA^10^. Some success was achieved in PNA conjugates of the metal-free artificial ribonuclease tris(2-aminobenzimidazole) targeted to miRNA 20a, a member of the oncogenic miRNA 17–92 cluster^12^. Recently we developed metal-independent artificial ribonucleases — peptide-oligonucleotide conjugates (POCs) targeted to highly oncogenic *mmu*-miR-21(‘miRNases’), capable to cleave site-specifically this miRNA exclusively at G-X linkages, and demonstrated specific inhibition of this miRNA in lymphosarcoma cells and significant reduction of cell proliferation^11^. It was shown that cleavage specificity of the conjugates varied depending on the synthetic scheme: attachment of the peptide to oligonucleotide via either C- or N-terminus. Attachment of the peptide via C-terminus resulted in conjugates with G-X cleavage specificity^11^, whereas attachment via N-terminus yielded conjugates with Pyr-X specificity^16^. In addition, the cleavage specificity was also determined by the sequence of the peptide^16^.

Here we developed novel series of miRNases — conjugates of oligonucleotides and catalytic construction NH_2_-Gly(ArgLeu)_4_-^5′^TCAA^3′^ targeted to highly oncogenic miRNAs miR-21 and miR-17. We demonstrated the ability of these conjugates to efficiently cleave miRNA-targets at phosphodiester bonds in Pyr-X motives and to inhibit oncogenic miRNA in tumor cells due to both the ribonuclease activity of the conjugate which is significantly enhanced in the presence of RNase H and recruiting of intracellular RNase H.

## Results

### Design of miRNA-targeted POCs

MicroRNAs are difficult objects for cleavage by sequence-specific artificial ribonucleases due to their short length and specific sequence often lacking linkages that are sensitive to hydrolysis in the 3′-region. Pyr-X sequences are considered to be the most susceptible to cleavage both spontaneous and inducing. Therefore, the development of conjugates with Pyr-X cleavage specificity for destroying miRNA is of great interest.

The objective of this work was developing miRNases with new type of specificity capable to cleave miRNA site-selectively at phosphodiester bonds in Pyr-X linkages. The conjugates targeted miRNAs – miRNases - are built of two parts: oligonucleotide complementary to miRNA target and catalytic fragment which could be peptide^11,16^ or other constructions displaying ribonuclease activity. In this work to design pyrimidine-X specific miRNases, we use as catalytic construction conjugate of a short oligodeoxyribonucleotide TCAA and a peptide with alternating leucine and arginine residues. As it was shown earlier, this conjugate NH_2_-Gly(ArgLeu)_4_-TCAA was one the most effective artificial ribonuclease which exhibited Pyr-A cleavage specificity^17,18^. Having no complementarity with RNA substrates this short POC was shown to efficiently cleave various RNAs at all accessible U-A and C-A motives in non-sequence-specific manner with minor activity at G-X linkages^17^. High ribonuclease activity of this conjugate let us to assume that it can be used as a catalytic part in the POCs containing oligonucleotide complementary to miRNA.

**Figure 1:**
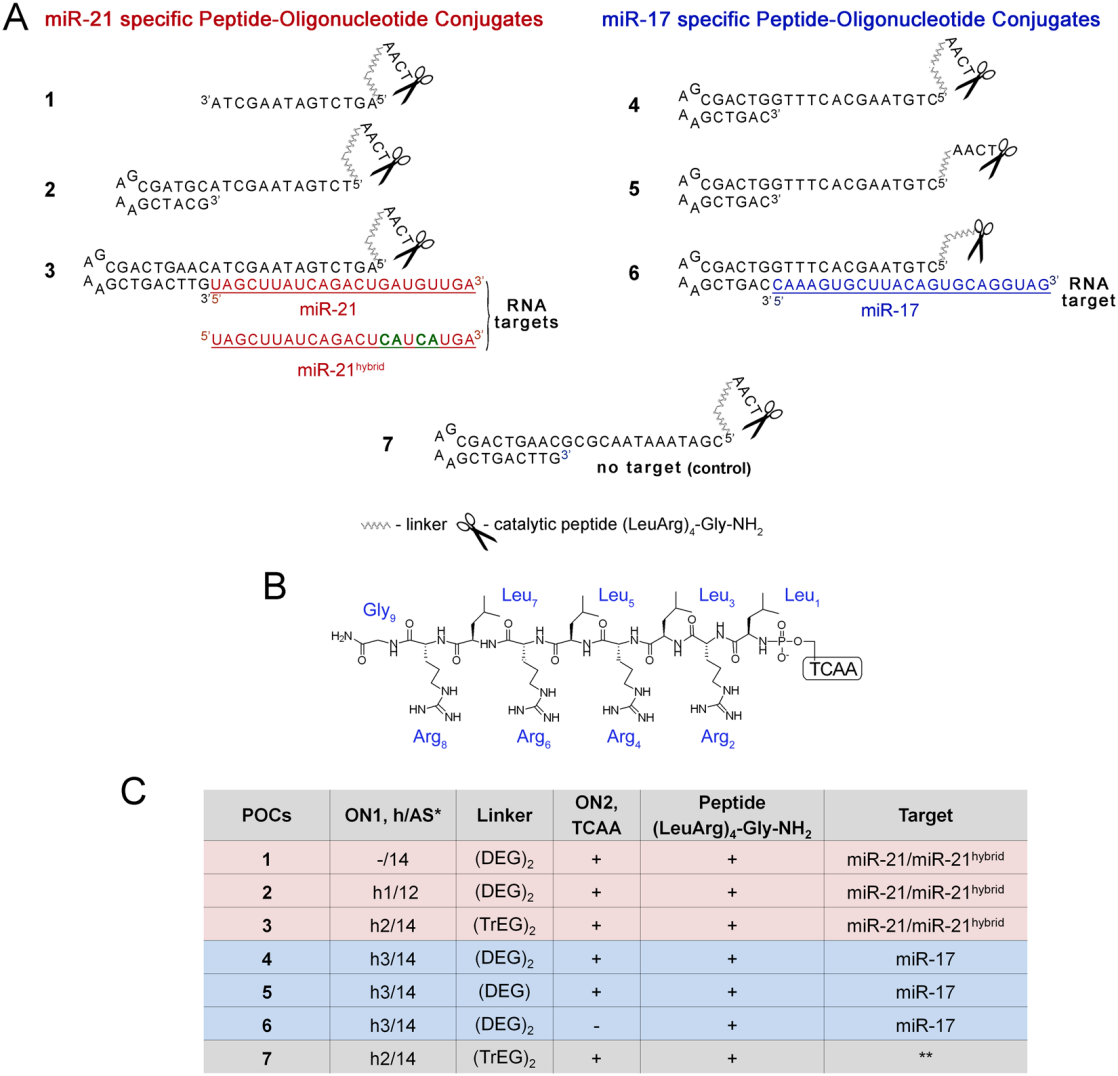
The structure of anti-miRNA peptide-oligonucleotide conjugates (POCs). (**A)** Schematic representation of a general complex of RNA target and peptide-oligonucleotide conjugate. (**B)** The chemical structure of the peptide (LeuArg)_4_-Gly-NH_2_. The peptide was conjugated via the N-terminus to the short oligonucleotide TCAA, which was attached via diethylene glycol (DEG) or tetraethylene glycol (TrEG) linker to the 5′-terminal phosphate of the antisense oligonucleotide. (**C)** Structural components of conjugates. Designations: ON1, h/AS – oligonucleotide in the conjugate containing hairpin (h) and sequence complementary to miRNA (AS); h1 and h3 – hairpins with 6 b.p. stem, h2 - hairpin with 9 b.p. stem; DEG – diethylene glycol; TrEG - tetraethylene glycol; ON2 – TCAA; * - conjugate 7 (control conjugate) was targeted to luciferase mRNA.

Novel POCs were designed as follows: conjugates consist of miRNA targeting oligonucleotide and catalytic construction ^C-terminus^NH_2_Gly(ArgLeu)_4_^N-terminus^-^5′^pTCAA^3′^; catalytic construction is attached to 5′-end of targeting oligonucleotide using long flexible linkers on the base of di- or tetraethylene glycol residues (Fig. 1A, Table 1). In the catalytic construction short oligonucleotide pTCAA is attached to the N-terminus of the peptide ^N-terminus^(LeuArg)_4_GlyNH_2_^C-terminus^ via a phosphoramidate linkage formed between 5′-phosphate of oligonucleotide and NH_2_ group of first leucine residue. MicroRNA-targeting oligonucleotides were either linear (conjugate **1**, Table 1, Fig. 1A) or have hairpin structure (conjugates **2–7**, Table 1, Fig. 1A) with the length of sequence complementary to miRNA 12–14 nts. Oligonucleotide sequences were chosen to cover seed regions of miRNAs upon binding. Hairpin oligonucleotides with 5 nt purine rich loop and 6–9 b.p. stem were used to provide better miRNA binding due to complementary complex stabilization by stacking interactions^19,20^. Also, in biological media hairpin structure protects oligonucleotide against exonuclease attack even without the use of additional chemical modifications^21–25^.

**Table 1:** Sequence, structure, and characterization of conjugates.

Name	Conjugate structure and oligodeoxyribonucleotide sequence (5′ → 3′)	Calculated [M-H]^−^ m/z	Found [M-H]^−^ m/z
1	*NH*_2_*—Gly(ArgLeu)*_4_*—P—TCAA*—(DEG)_2_—**AGT CTG ATA AGC TA**	7056.2	7058.39
2	*NH*_2_*—Gly(ArgLeu)*_4_*—P—TCAA*—(DEG)_2_—**TCT GAT AAG CTA** CGT AGC GAA AGC TAC G	11479.07	11480.68
3	*NH*_2_*—Gly(ArgLeu)*_4_*—P—TCAA*—(TrEG)_2_—**AGT CTG ATA AGC** TAC AAG TCA GCG AAA GCT GAC TTG	14062.77	14062.21
4	*NH*_2_*—Gly(ArgLeu)*_4_*—P—TCAA*—(DEG)_2_—**CTG TAA GCA CTT T**GG TCA GCG AAA GCT GAC	12001.52	12002.83
5	*NH*_2_*—Gly(ArgLeu)*_4_*—P—TCAA*—(DEG)—C**TG TAA GCA CTT TG**G TCA GCG AAA GCT GAC	11833.43	11835.04
6	*NH*_2_*—Gly(ArgLeu)*_4_—P— (DEG)_2_—**CTG TAA GCA CTT TG**G TCA GCG AAA GCT GAC	10781.71	10782.85
7	*NH*_2_*—Gly(ArgLeu)*_4_*—P—TCAA*—(TrEG)_2_—**CGA TAA ATA ACG CG**C AAG TCA GCG AAA GCT GAC TTG	14056.78	14055.80

Bold font - miRNA recognizing oligonucleotide tracts; underline font - hairpin; italic font -catalytic part. DEG and TrEG: di- and tetraethylene glycol. The sequences of oligonucleotides **1** and **3** are consistent with the sequences of oligonucleotides of corresponding conjugates.

Oncogenic miR-21 and miR-17 associated with different types of cancer were chosen as targets. These miRNAs which participate in regulation of cell cycle and apoptosis, enhance proliferation of tumor cells and stimulate metastasizing^26–28^, represent attractive targets for antisense-based therapeutics.

RNA cleaving properties of designed POCs, as well as the influence of the linker structure and flexibility on their catalytic activity and specificity of cleavage were studied using two series of the conjugates: (i) POCs targeted to miR-21, and (ii) POCs targeted to miR-17 (Fig. 1A,C). In the series (i) (conjugates **1**, **2** and **3**) the catalytic structure remains invariable and was connected through double DEG (diethylene glycol) or double TrEG (tetraethylene glycol) to targeting oligonucleotides differed by length and structure: conjugates contain a sequence complementary to miR-21 of 12 (conjugate **2**) or 14 nt (conjugates **1** and **3**, Fig. 1) in length. DEG and TrEG-based linkers were chosen to increase the flexibility of the catalytic group upon formation of complementary complex with target miRNAs. It has been demonstrated previously that the use of a DEG-based linker for peptide attachment to long oligonucleotides allows one to obtain an advantage in the cleavage efficiency of RNA target^29^. The conjugates **2** and **3** contain hairpin oligonucleotides with 6 (hairpin h1) or 9 (hairpin h2) b.p. stem (Fig. 1, Table 1). In the series (ii) (conjugates **4**, **5** and **6**) there was 14 nt sequence complementary to miR-17 and hairpin h3 with 6 b.p. stem (Fig. 1, Table 1). Oligonucleotides were attached to the catalytic domain through double DEG or single DEG linkers (conjugates **4** and **5**, Fig. 1, Table 1). The conjugate **6** lacks the short oligonucleotide TCAA within the catalytic construction and the peptide was attached directly to the addressing oligonucleotide via double DEG linker (Fig. 1). Сontrol conjugate **7** addressed to luciferase mRNA was synthesized. This conjugate contains 14 nt targeted sequence, hairpin 2 and catalytic domain similar to all other conjugates attached to 5′-terminal phosphate group of oligonucleotide via double tetraethylene glycol linker (Fig. 1).

### Synthesis and stability of the conjugates

Synthesis of the starting oligonucleotides was carried out by stepwise condensation of standard synthons by the phosphoramidite protocol. The introduction of non-nucleotide fragments (5′-phosphate, linker groups) was carried out during the synthesis of the oligonucleotide according to recommendation of synthons manufacturer. Conjugation of oligonucleotides with the peptide was performed by using Mukaiyama conditions^30^: a redox pair of triphenylphosphine – 2,2′-dipyridyl disulfide due to formation an intermediate phosphoroxyphosphonium salt was used as a coupling reagent in the presence of DMAP as a nucleophilic catalyst. When the terminal phosphate group is activated, the derivative of the oligonucleotide reacts rapidly with amines over a wide range of p*K*_*a*_, and with increasing p*K*_*a*_, the rate of formation of the phosphoamide bond increases. In the attached peptide, the guanidinium group of arginine is the most basic, but when using the peptide as a trifluoroacetate salt, this group remains protonated under the reaction conditions and the terminal α-amino group of the peptide interacts with the activated phosphate group of the oligonucleotide. The attachment of a peptide fragment to an oligonucleotide changes the chromatographic mobility of this molecule (the retention time), so that the degree of conversion as a measure of the relative yield of the conjugate can be evaluated by the ratio of the areas of the initial peak and the peak of the product on the chromatogram. The conversion level when applying this method was 85–90%. The average yield of the conjugates after synthesis and purification by RP-HPLC was 60–75% with respect to initial amount of starting oligonucleotides.

Identity and purity of the synthesized POCs have been confirmed by RP-HPLC, urea-PAGE, MALDI spectroscopy, and ESI-MS (Fig. S1–S3, Supplementary Information). The attachment of the peptide to the oligonucleotide results in an increase in its hydrophobicity, which is recorded by RP-HPLC as an increase of retention time of the reaction product on the column as compared to the starting oligonucleotide. As expected the introduction of a positively charged peptide reduced the electrophoretic mobility of the conjugates, as compared to the starting oligonucleotides. The masses of the obtained conjugates were consistent with the theoretically calculated ones (Table 1).

**Figure 2:**
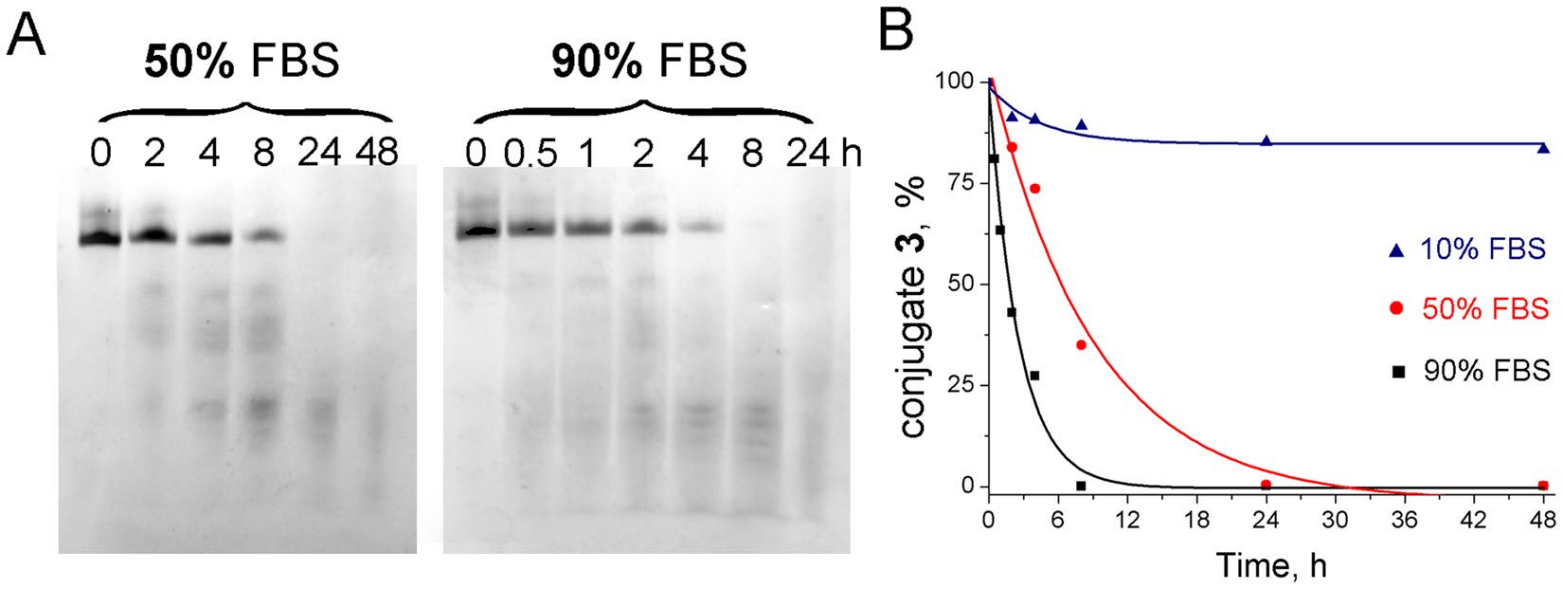
Stability of conjugate **3** in DMEM supplemented with 10–90% fetal bovine serum. (**A)** Stability of the conjugate **3** in DMEM supplemented with 50% (left panel) and with 90% FBS (right panel). Autoradiographs of 12% polyacrylamide/8 M urea gel. (**B)** The time course of conjugate **3** degradation in 10 (triangles), 50 (circles) and 90% (squares) FBS.

The study of stability of oligonucleotides and conjugates was performed with the example of oligonucleotide **1** which sequence was consistent with the sequence of oligonucleotide in conjugate **1**, oligonucleotide **3** which sequence was consistent with the sequence of oligonucleotide in conjugate **3**, and conjugate **3**. The study of stability revealed that in medium with 10% fetal bovine serum the half-life time for linear oligonucleotide **1** is less than 1 h whereas the introduction of the hairpin with an elongated stem to the 3′-end of oligonucleotide (oligonucleotide **3**) resulted in the increase of its half-life time to 48 h. Attachment of a catalytic construction to the 5′-end of a hairpin oligonucleotide (conjugate **3**) resulted in essential increase of conjugate stability: only 20% of hydrolysis in serum-containing medium was observed at 48 h time point (Fig. 2). In the presence of 50% FBS conjugate **3** kept their stability up to 24 h, and in the presence of 90% FBS – up to 8 h (Fig. 2), whereas corresponding oligonucleotide **3** was totally hydrolyzed after 10 min of incubation (primary data not shown).

### Cleavage of non-complementary RNA substrates by conjugates

Short oligonucleotide-peptide conjugate NH_2_-Gly(ArgLeu)_4_-TCAA used as RNA-cleaving domain within POCs exhibited high ribonuclease activity when used alone. The addition of the miRNA-targeted antisense oligonucleotide to this catalytic construction could affect its ribonuclease activity and sequence-specificity. Given this we analyzed the cleavage of the 96 nts fragment of HIV-1 RNA having pronounced secondary structure by the conjugate **3** which does not contain any complementary sites in this RNA and compared it with published data on cleavage of the same RNA by the conjugate NH_2_-Gly(ArgLeu)_4_-TCAA.

It was found that the designed miRNA-specific conjugate **3** efficiently cleaves HIV-1 RNA, exhibiting predominantly Pyr-A cleavage specificity (Fig. S4A,B, Supplementary information). The kinetics of RNA cleavage showed that almost total cleavage of HIV-1 RNA is achieved within 24 h. At this time point 85% of RNA is already cleaved and longer incubation leads to accumulation of the shortest 5′-[^32^P]-labeled fragment correlated to the cleavage at U_7_-A_8_ linkage (Fig. S4A,B,D), thus showing that during all this time conjugate **3** remained active and worked as a ribonuclease. Analysis of cleavage products showed that the pattern of RNA cleavage by conjugate **3** coincides with that observed earlier for NH_2_-Gly(ArgLeu)_4_-TCAA^17,18^.

Further, the ribonuclease activity of the conjugate **3** was also examined with the use of a shorter and unstructured non-complementary substrate — miR-17. Analysis of miR-17 cleavage showed that conjugate **3** cleaves miR-17 with similar efficiency, as HIV-1 RNA — 90% of RNA was cleaved during 24 h (Fig. S4C,D). The main linkages that undergo cleavage were also within C-A and U-A motives. Thus, attachment of miRNA-targeted oligonucleotide via di-tetraethylene glycol linker does not alter the ribonuclease activity of conjugate NH_2_-Gly(ArgLeu)_4_-TCAA neither in terms of cleavage efficiency, nor in cleavage specificity.

### Cleavage of miRNA-targets by miRNA-specific conjugates

The ability of the designed POCs **1**–**6** to cleave site-selectively miRNAs was studied using three RNA substrates that might differ in sensitivity to the developed type of conjugates: (1) miR-21, in which the 3′-region (5′-_15_GAUGUUGA_22_-3′) available for cleavage by the conjugate does not contain U-A and C-A bonds, the most sensitive for this type of conjugates; (2) miR-21^hybrid^, in which an alterations in the sequence of the 3′-region were introduced, so that this region carries two C-A bonds (5′-_15_**CA**U**CA**UGA_22_-3′); and (3) miR-17, the 3′-region of which contains C-A and U-A motives (5′-_15_UG**CA**GG**UA**G_23_-3′) (Fig. 1A).

#### Cleavage of miR-21 by conjugates 1, 2, and 3

**Figure 3:**
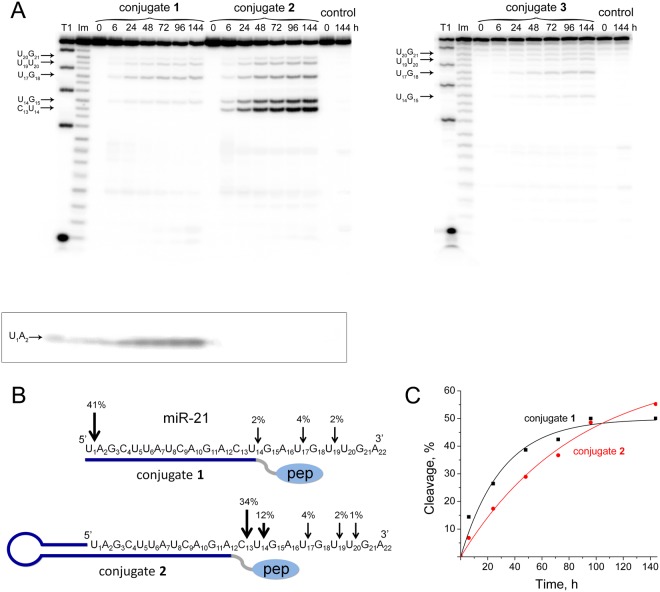
Cleavage of 5′-[^32^P]-miR-21 by conjugates **1**, **2**, and **3**. (**A)** Autoradiograph of 18% polyacrylamide/8 M urea gel, showing the pattern of miR-21 cleavage by the conjugates. Lanes Im and T1 — imidazole ladder and partial RNA digestion with RNase T1, respectively; control — RNA incubated in the absence of conjugates for 0 and 144 h. miR-21 (1 µM) and conjugates (100 µM) were incubated at 37 °C for 0–144 h. The conjugate type and incubation time are shown at the top. The image enclosed by lines is part of the upper left image but scanned as separate file. (**B)** Positions of miR-21 cleavage by conjugates **1** and **2**. The percentage of cleavage at specific sites in 144 h is indicated above the arrows; pep — catalytic construction. (**C**). Kinetics of miR-21 cleavage by conjugates **1** and **2**.

The cleavage of miR-21 by the conjugates **1**, **2** and **3**, differing in length and structure of the addressing oligonucleotide, shows that, as expected, this target demonstrates high stability in the presence of miRNases. At a 20-fold excess of conjugates, the cleavage of miRNA-target does not exceed 1% for conjugate **3** and 3–4% for conjugates **1** and **2** after 72 h of incubation (primary data not shown). With an increase in the concentration of the conjugate to 100-fold excess — 100 µM — concentration at which some unspecific interaction could take place, the extent of cleavage increases (Fig. 3A).

It can be seen that the efficiency of the conjugates **1**, **2** and **3** significantly differs. The conjugate **3** cleaves miR-21 very slightly even at a 100-fold excess (Fig. 3A). The conjugates **1** and **2** in a 20-fold excess cleave miR-21 by no more than 4%, while increasing the concentration to a 100-fold excess increases the cleavage efficiency of the target to 50% (Fig. 3A,C). It is evident that the different structure of oligonucleotide part of conjugates dictates the pattern of miR-21 cleavage. The conjugate **1** cleaves miR-21 predominantly at the U_1_-A_2_ linkage, which located at the 5′-end of the molecule and is the most distant from the site of attachment of the catalytic domain (Fig. 3A,B). The linear oligonucleotide of the conjugate **1** forms obviously a breathing duplex with miRNA so that in the absence of the hairpin structure, flanking the 5′-end of miRNA, the site U_1_-A_2_ of miR-21 becomes apparently available for cleavage. The conjugate **2** cleaves miR-21 at the 3′-region predominantly at the C_13_**-**U_14_ and U_14_**-**G_15_ linkages and to a much lesser degree at the linkages U_17_-G_18_, U_19_**-**U_20_ and U_20_**-**G_21_ at the very end of miRNA molecule (Fig. 3A,B). It is evident that upon cleavage of miR-21 the conjugates with an oligonucleotide moiety of 14 nucleotides in length (conjugates **1** and **3**) do not work effectively, whereas shortening of the complementary sequence up to 12 nucleotides (conjugate **2**) significantly increases the efficiency of site-specific cleavage by the conjugates. It is apparent that the shorter complementary part (12 nt) uncovers additional more sensitive sites of the target RNA to attack by the conjugate, in this particular case it is C_13_**-**U_14_ linkage. The performed experiments revealed an important result – the engineered conjugates are capable of cleaving the target not only at C-A and U-A sites, but in the absence of more sensitive motives at C-U, U-G and U-U linkages, which are known to be more stable for cleavage^31^. Thus, using the designed conjugates, for the first time it was possible to perform the cleavage of RNA, in particular miRNA, at C-U, U-G and U-U phosphodiester bonds, however efficiency of this process is rather low.

#### Cleavage of miR-21^hybrid^ by conjugates 1, 2, and 3

**Figure 4:**
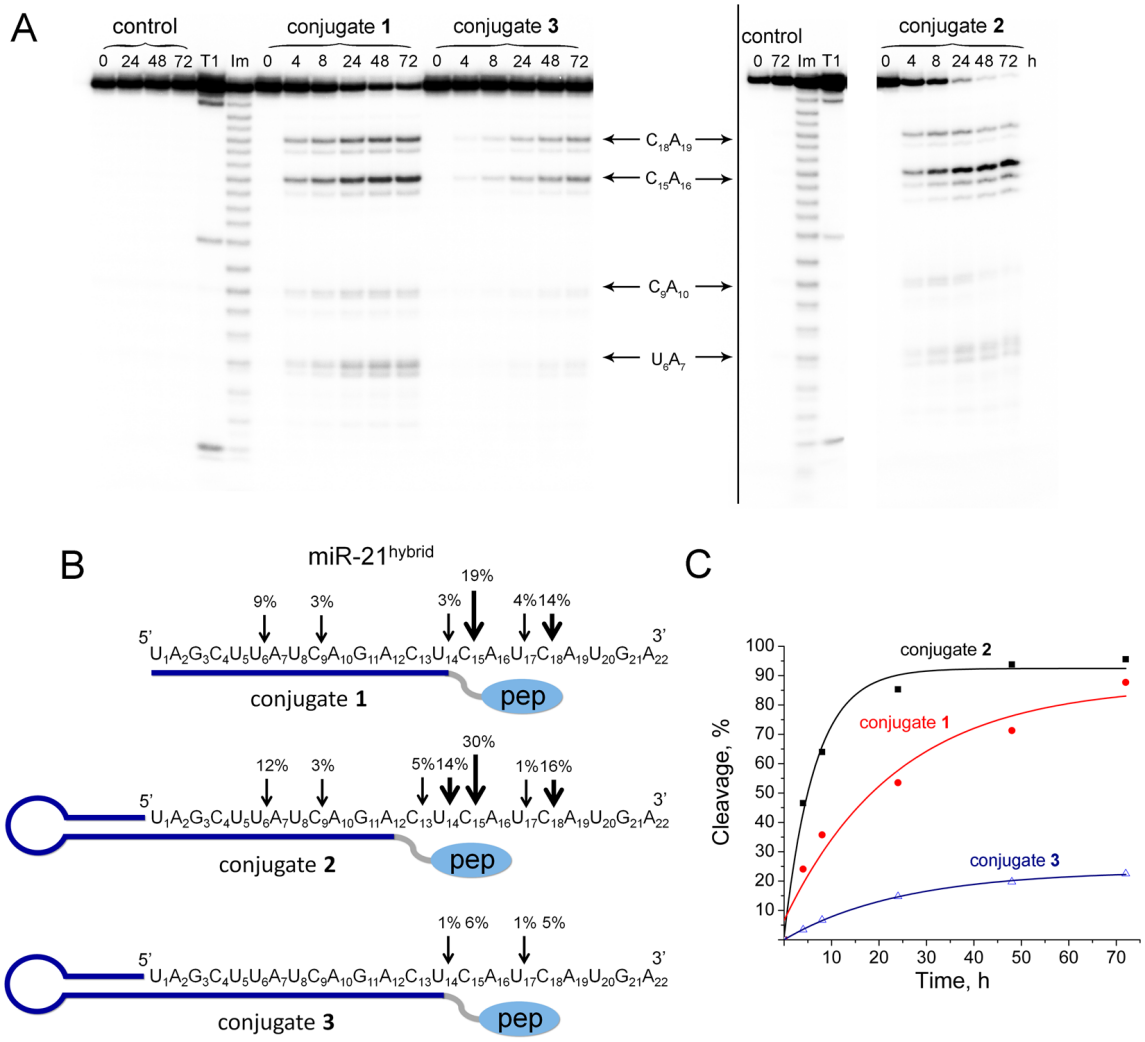
Cleavage of 5′-[^32^P]-miR-21^hybrid^ by conjugates **1**, **2**, and **3**. (**A)** Autoradiograph of 18% polyacrylamide/8 M urea gel showing the pattern of miR-21^hybrid^ cleavage by the conjugates. Lanes Im and T1 — imidazole ladder and partial RNA digestion with RNase T1, respectively; control — RNA incubated in the absence of conjugates for 0–72 h. miR-21 (1 µM) and conjugates (20 µM) were incubated at 37 °C for 0–72 h. The conjugate type and incubation time are shown at the top. The images enclosed by lines are parts of the same gel. (**B)** Positions of miR-21^hybrid^ cleavage by conjugates. The percentage of cleavage at specific sites in 24 h is indicated above the arrows; pep — catalytic construction. (**C**). Kinetics of miR-21^hybrid^ cleavage by conjugates.

Similar experiments performed with miR-21^hybrid^ have shown that, according to expectations, the presence of two C-A bonds at the 3′-region of miR-21 significantly increased the sensitivity of miRNA-target to cleavage by the conjugates (Fig. 4). As soon as two C-A motives appears in the 3′-region of miR-21 the total extent of miR-21^hybrid^ cleavage at a 20-fold excess of conjugates significantly increases from 1–4% up to 55, 85 and 12% (24 h time point) for the conjugate **1**, **2**, and **3**, respectively (Fig. 4C).

The designed miR-21^hybrid^ is predominantly cleaved at C-A linkages specially introduced in the 3′-region (Fig. 4A,B). The presence of C-A sites near the location of the catalytic peptide upon the formation of the duplex with miRNA completely prevents the cleavage of the U_1_A_2_ bond by the conjugate **1**, which was revealed for the miR-21 target. For the conjugates **1** and **2** the appearance of products of secondary cleavage at C_9_-A_10_ and U_6_-A_7_ sites of miR-21^hybrid^ was observed. The presented data clearly indicate that among the conjugates studied, conjugate **2** with a hairpin oligonucleotide and a complementary sequence of 12 nucleotides demonstrates the highest cleaving efficiency of both miR-21 and miR-21^hybrid^ and is an effective pyrimidine-specific miRNase.

#### Cleavage of miR-17 by conjugates 4, 5, and 6

**Figure 5:**
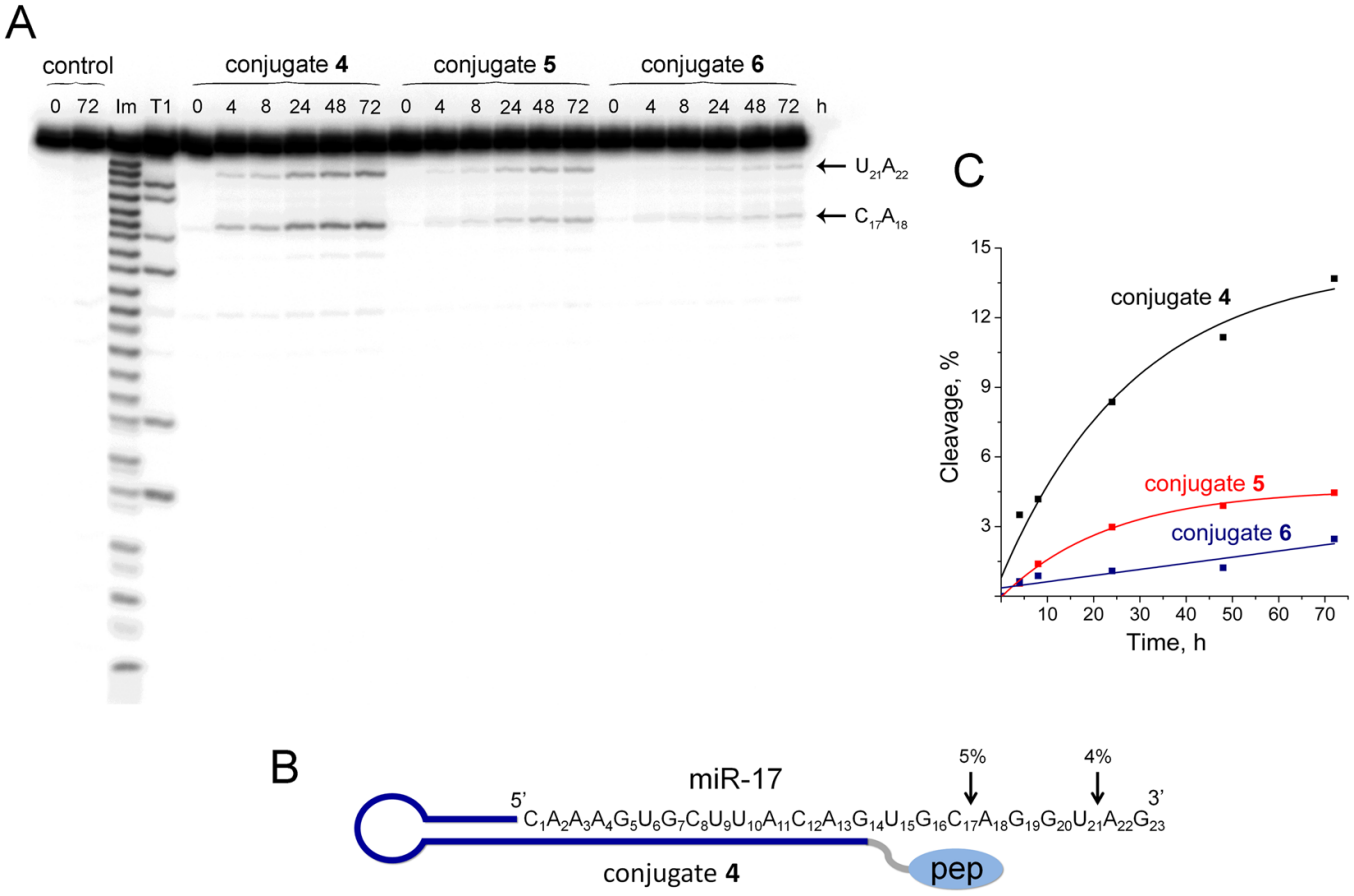
Cleavage of 5′-[^32^P]-miR-17 by conjugates **4**, **5**, and **6**. (**A)** Autoradiograph of 18% polyacrylamide/8 M urea gel showing the pattern of miR-17 cleavage by the conjugates. Lanes Im and T1 — imidazole ladder and partial RNA digestion with RNase T1, respectively; control — RNA incubated in the absence of conjugates for 0 and 72 h. miR-17 (1 µM) and conjugates (20 µM) were incubated at 37 °C for 0–72 h. The conjugate type and incubation time are shown at the top. The images enclosed by lines are parts of the same gel. (**B)** Positions of miR-17 cleavage by conjugate **4**. The percentage of cleavage at specific sites in 24 h is indicated above by arrows; pep — catalytic construction. (**C**). Kinetics of miR-17 cleavage by conjugates.

The activity of the designed type of conjugates was tested with another RNA model — miR-17. The experiments were carried out with the use of conjugate **4**, which has some common features with conjugate **2**, but differ from it by the length of hairpin stem (6 b.p. in **2** and 9 b.p. in **4**) and miRNA-complementary sequence (12 nt in **2** and 14 nt in **4**). The cleavage assay showed that the conjugate **4** at a 20-fold excess cleaves miR-17 by 9% after 24 h and demonstrates the same cleaving efficiency as conjugate **3** in experiments with miR-21^hybrid^ (Fig. 5A,B). The cleavage occurs site-specifically at two sites in the 3′-region of miR-17 — C_17_**-**A_18_ and U_21_**-**A_22_ (Fig. 5A,C). The study of the influence of the linker length and structure on ribonuclease activity showed that shortening of the linker to one DEG in the conjugate **5** leads to significant decrease in ribonuclease activity of the conjugate. The efficiency of the cleavage dropped to 3% (Fig. 5A,B). More interestingly that the deletion of the short oligonucleotide TCAA from the catalytic construction in conjugate **6** leads to complete loss of ribonuclease activity (Fig. 5A,B). Thus, it is clear that double DEG linker provides sufficient flexibility of the catalytic construction, and a short oligonucleotide TCAA is required as an important structural element of the artificial ribonuclease, likely providing catalytically active tertiary structure of the peptide.

The data obtained clearly show that the efficiency of the conjugates directly depends on the sequence of miRNA-target and especially RNA fragment intended for cleavage. The designed conjugates cleave RNA targets exclusively after the residues of the pyrimidines, and the reactivity to phosphodiester bonds formed by different base pairs significantly differs. The sensitivity of phosphodiester bonds to the cleavage by this type of conjugates can be arranged in the following order: U-A = C-A ≥ C-U ≥ U-G = U-C. In addition, the sensitivity of the phosphodiester bonds to cleavage also depends on the neighboring nucleotides adjacent to the phosphodiester bond at the 3′- and 5′-end and their location inside the RNA-target.

### Hybridization efficiency of conjugates 1, 2, and 3 with miR-21

**Figure 6:**
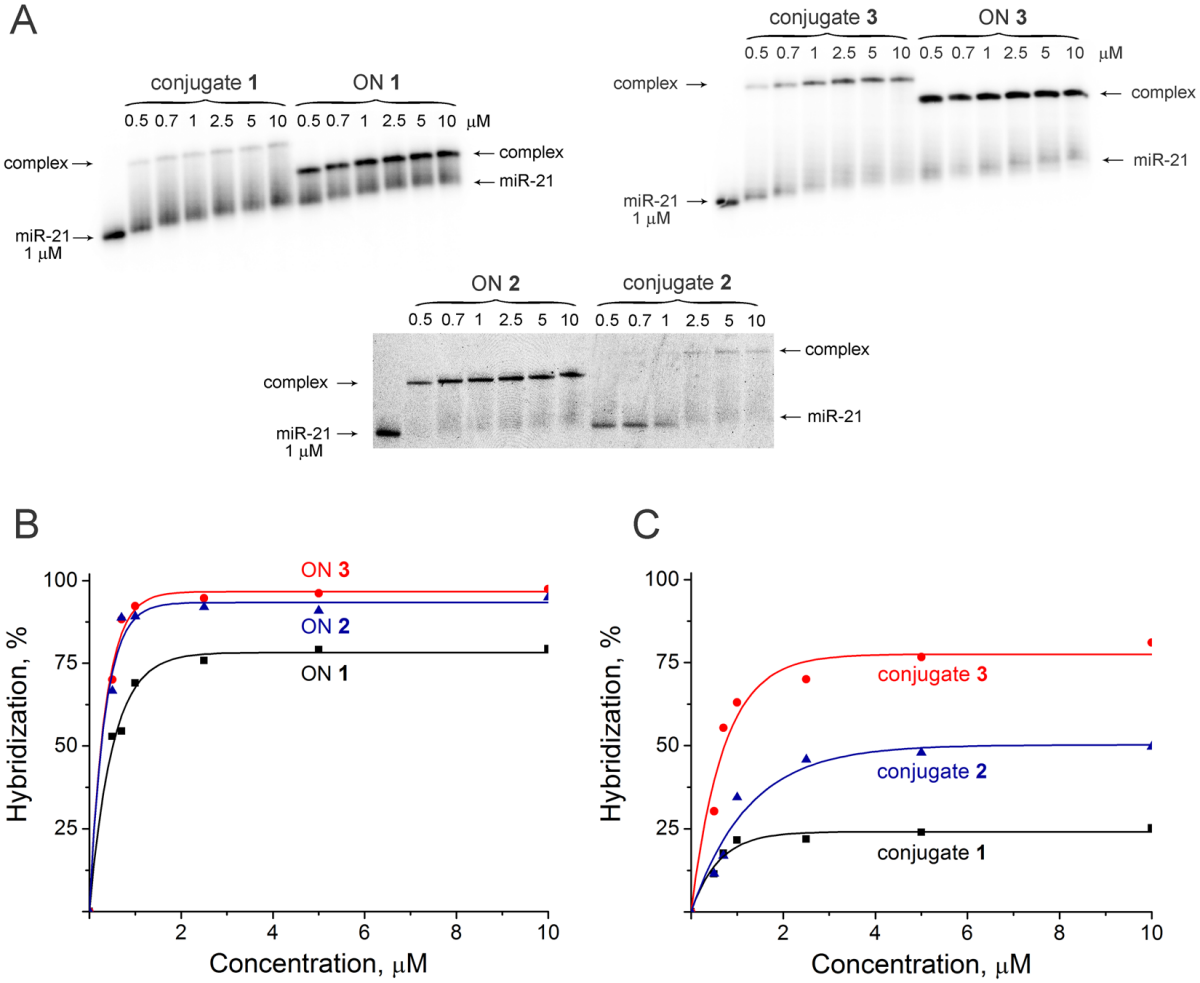
Hybridization efficiency of conjugates **1**–**3** and corresponding oligonucleotides **1**–**3** (ON **1**–**3**) with 5′-[^32^P]-miR-21. (**A)** Autoradiograph of 15% native PAGE. (**B**,**C)**. Concentration dependencies of binding of oligonucleotides and conjugates with miR-21.

In order to evaluate how the hybridization properties of conjugates correlate with the ribonuclease activity, the binding ability of conjugates **1**, **2**, and **3** was assessed and compared with the binding ability of corresponding oligonucleotides unconjugated with a peptide. Gel retardation assay and concentration analysis of complex formation showed that oligonucleotide **3** and conjugate **3** in which the oligonucleotide moiety contained 14 nt sequence complementary to miR-21, and 9 b.p. stem, bound to miR-21 most efficiently: at equimolar concentration and higher, the hybridization efficiencies were 96 and 77%, respectively (Fig. 6).

The binding efficiency of oligonucleotide **2** containing the oligonucleotide moiety with 6 b.p. stem and 12 nt sequence complementary to miR-21 was close to the binding efficiency of oligonucleotide **3.** Nevertheless, conjugate **2** bound to miR-21 less effectively and binding efficiency reached only 48% (Fig. 6), that can be associated both with shorter stem and sequence complementary to miR-21.

The binding efficiency of oligonucleotide **1** to miR-21 was 75%, whereas the binding of conjugate **1** to miR-21 was significantly weaker than the corresponding oligonucleotide: in an equimolar concentration and higher, the percentage of binding was only 25% (Fig. 6). Hence, the presence of a hairpin in conjugates **2** and **3** significantly enhanced its binding capacity as compared to linear conjugate **1**. These data well correlate with thermodynamic parameters of these oligonucleotides. For a 14-mer oligonucleotide **1** Tm of the duplex with miRNA target is 47.8 °C^20^, and Tm of the duplex formed by oligonucleotide **2** with miRNA is 41.7 °C^20^. For oligonucleotide **3** the stabilizing effect provided by cooperative interactions at the junction of the stem of the hairpin structure and duplex formed by oligonucleotide with RNA is observed. This is reflected in the increase in Tm of a heteroduplex to 53.3 °С^20^.

With regard to ribonuclease activity, it can be concluded that the formation of the complex significantly slowed down the work of conjugate **3** and conjugate **2**, providing the dynamics of complex formation, which, while maintaining site-specificity, provided an increase in the efficiency of miRNA cleavage.

### Biological effects of conjugate 4 in melanoma B16 cells

The biological activity of the developed type of conjugates was studied using melanoma B16 cells and conjugate **4** targeted to miR-17. Conjugate **7** targeted to the luciferase mRNA region, the sequence of which was not found in the mammalian genome, was used as a control. Similarly, oligonucleotide **4** was used in this experiment to check advantages of conjugate **4**.

Murine melanoma cells B16 were transfected with anti-miR-17 conjugate **4**, antisense oligonucleotide **4**, or control conjugate **7** taken in the concentration range 0.05–1 µM and precomplexed with Lipofectamine^TM^ 2000.

#### Effect of conjugate 4 on the growth rate of melanoma cells

**Figure 7:**
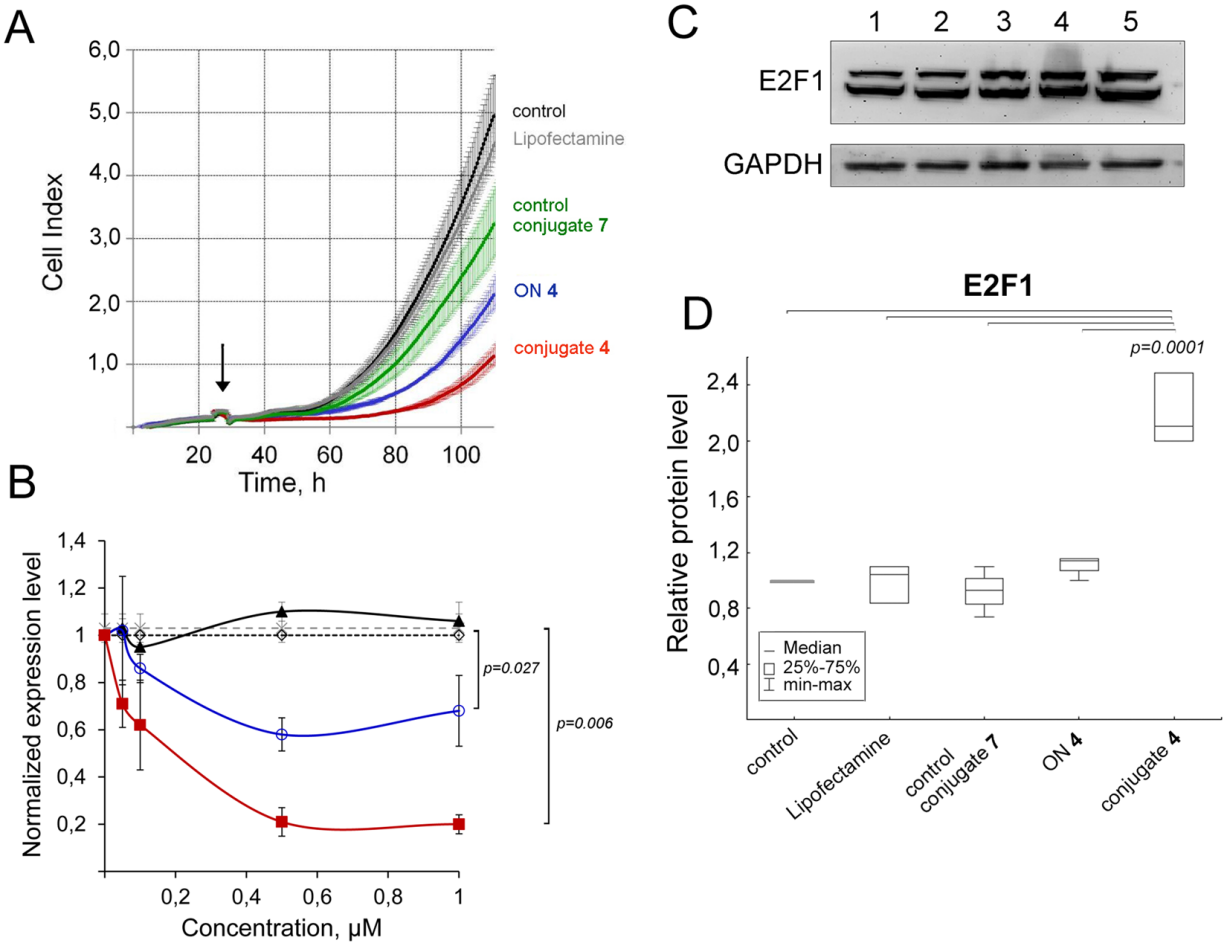
Biological effect of conjugate **4** in melanoma B16 cells. (**A)** Real-time analysis of the effect of anti-miR-17 conjugate **4** on the growth rate of B16 melanoma cells. B16 melanoma cells were transfected with conjugate **4**, control conjugate **7**, oligonucleotide **4** (ON **4**) precomplexed with Lipofectamine 2000 at a concentrations of 1 µM. B16 cells without any treatment and B16 treated with Lipofectamine were used as controls. Transfection time is indicated by arrow. The data were statistically processed using the Student’s t-test (two-tailed, unpaired). The results are shown as mean cell index ± standard error. (**B)** Expression level of miR-17 in tumor cells after transfection with anti-miR-17 conjugate **4**. Control conjugate **7**, conjugate **4** and oligonucleotide **4** (ON **4**) were transfected in complex with Lipofectamine 2000 into melanoma cells B16 in concentration range 0.05–1 µM. In 24 h after transfection RNA was isolated and stem-loop PCR was performed. The expression of miR-17 was normalized to U6. Data were statistically analyzed using one-way ANOVA with post hoc Tukey test. Data are given as mean calculated from three independent experiments ± SEM. Open diamond symbol — control, cells without any treatment; cross — cells treated with Lipofectamine; close triangle — cells treated with control conjugate **7**; open circle — cells treated with oligonucleotide **4**; close square — cells treated with conjugate **4**. (**C)** Western blot analysis of E2F1 protein level 72 h after transfection. GAPDH served as an internal control. 1 **─** intact melanoma B16 cells; 2 **─** melanoma B16 cells incubated with Lipofectamine 2000; 3, 4, 5 **─** melanoma B16 cells incubated with 1 µM control conjugate **7**, antisense oligonucleotide ON **4** and anti-miR-17 conjugate **4**, respectively. (**D)** The bar graph shows the semi-quantitative analysis of the Western blot results for E2F1. Data were statistically analyzed using one-way ANOVA with post hoc Tukey test.

The effect of anti-miR-17 conjugate **4** on proliferation of B16 cells was monitors in real time (Fig. 7A). In the absence of any treatment melanoma B16 cells intensely proliferated for 120 h so that their population increased fivefold (Fig. 7A), then the population size started to decrease, and the mean cell index was 5 (Fig. 7A). Transfection of B16 cell with Lipofectamine has no effect on their growth rate, and proliferation index was similar to index of control cells. Control conjugate **7** slightly decreased cell index to 3.2, however, this difference was statistically insignificant. Antisense oligonucleotide **4** caused 2.5-fold decrease of growth rate, and cell index was 2 (Fig. 7A). Conjugate **4** had the most pronounced effect on proliferation of melanoma cells causing 5-fold inhibition of cell proliferation; cell index was 1 (Fig. 7A).

#### Effect of conjugate 4 on the level of miR-17

The level of miR-17 was evaluated at 24 h after transfection using stem-loop qPCR. Analysis of qPCR data shows that in 24 h post transfection, conjugate **7** does not evoke any statistically significant reduction in the miR-17 level (Fig. 7B). Anti-miR-17 oligonucleotide **4** leads to a 40% decrease in the miR-17 level: statistically significant reduction is observed at concentrations of 0.5 and 1 μM of the oligonucleotide. The most pronounced effect was observed for anti-miR-17 conjugate **4**: statistically significant reduction of miR-17 level starts from 0.1 µM of conjugate **4** and reaches maximum (80% reduction) at concentrations of 0.5 and 1 µM of the conjugate (Fig. 7B).

In order to confirm the absence of noticeable off-target effect for the developed conjugates, we examined the expression levels of let 7-g, miR-21 and miR-18a, which do not contain sequences complementary to those of the designed conjugates (Fig. S5). In this experiment cells were incubated with conjugate **4** for 24 h, since the most significant decrease in miR-17 level caused by anti-miR-17 conjugate **4** was observed at this time point. From the data shown in Fig. S5 it is seen that there are no statistically significant differences between the test samples and the controls. All observed deviations lay within the statistically acceptable error. These data well correlated with our data obtained recently showing the absence of off-target effects of miR-21-targeted conjugate of the similar type^11^.

#### The effect of conjugate 4 on the level of miRNA-17 protein target E2F1

The decrease in the level of oncogenic miR-17 in tumor cells caused by the conjugate **4** should promote the restoration of normal activity of tumor-suppressive target genes of miR-17 and as a consequence their protein products. miR-17 protein target E2F1 is involved in the regulation of cell cycle and activity of tumor suppressor proteins. Moreover, there are some evidences that E2F1 exhibit complex effect on melanoma cells controlling targets associated with primary or metastatic phenotype, such as hTERT and ASK/Dbf4^32^. In order to determine if there was any alteration in the level of protein E2F1, the direct target of miR-17, Western blot analysis was performed 72 h after transfection of B16 cells with 1 μM of control conjugate **7**, antisense oligonucleotide **4** and conjugate **4** (Fig. 7C,D). The data obtained show that there is no statistically significant change in the level of E2F1 in tumor cells incubated with Lipofectamine, control conjugate **7** and antisense oligonucleotide **4**, while in the cells incubated with antimiR-17 conjugate **4**, when the reduction in miR-17 level is observed, the level of E2F1 protein increases 2.2-fold as compared with control (Fig. 7C).

### Cleavage of miR-17 by combination of miR-17-specific POC and RNase H

**Figure 8:**
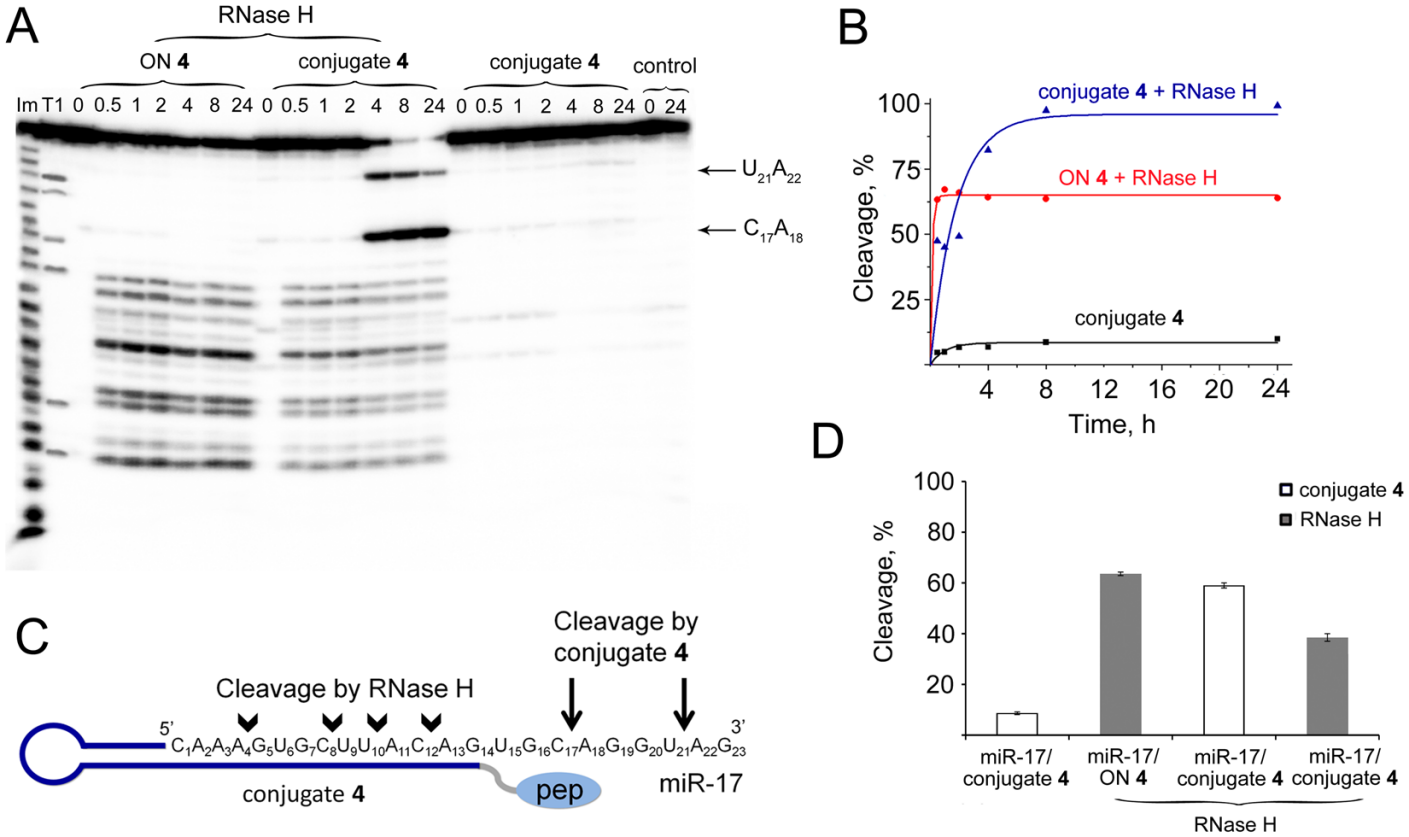
Cleavage of miR-17 by miR-17-specific conjugate **4** and RNase H. (**A)** Patterns of 5′-[^32^P]-miR-17 cleavage in complex with conjugate **4** or in complex with oligonucleotide **4** (ON **4)** by RNase H. Autoradiograph of 18% polyacrylamide/8 M urea gel. Duplexes of 5′-[^32^P]-miR-17 (10 μM) and oligonucleotide or conjugate (5 μM) were incubated at 37 °C for 24 h with RNase H (100 U/ml). Lanes Im and T1 — imidazole ladder and partial RNA digestion with RNase T1, respectively; control — RNA was incubated in the absence of oligonucleotide/conjugate and in the presence of RNase H. (**B)** Kinetics of miR-17 cleavage by conjugates **4**, miR-17/ON **4** cleavage by RNase H, and miR-17 cleavage by conjugate **4** together with RNase H. (**C)** Positions of miR-17 cleavage by RNase H and conjugate **4**. Cleavage at specific sites is indicated by arrows; pep — catalytic construction. (**D)** Diagram showing contribution of conjugate **4** and RNase H in the total cleavage of miR-17 for 8 h. Dark grey bars and white bars show contribution of RNase H and conjugate **4** to the miR-17 cleavage, respectively.

It is well-known that one of the mechanisms of mRNA and miRNA suppression in cells by antisense technology is degradation of the target RNA in the DNA:RNA heteroduplex by intracellular RNase H. Since unmodified oligodeoxyribonucleotides were used for the construction of conjugates, the duplex formed by miRNA and the conjugate can be recognized in cells by RNase H. To evaluate the effect of RNase H on the cleavage of miRNA in complex with the conjugate, we studied the degradation of miR-17 in the complex with conjugate **4** in the presence of RNase H and compared it with miR-17 cleavage in heteroduplex with oligonucleotide **4**. The reaction was carried out under a two-fold excess of the target RNA with respect to the conjugate. It was shown that under these conditions in 24 h the level of miR-17 cleavage by conjugate **4** alone reached 9% (Fig. 8A–C). Incubation of the complex of miR-17 with the oligonucleotide in the presence of RNase H resulted in 65% degradation of the 5′-region of RNA after 30 min of incubation when a plateau was reached (Fig. 8A–C). Incubation of the complex of miR-17 with conjugate **4** in the presence of RNase H led to a manifold increase in the efficiency of site-specific cleavage of miRNA by the conjugate. During the first 2 h, the cleavage mainly occurred at the sites corresponding to cleavage by RNase H (compared with cleavage sites of RNase H in miR-17 in complex with oligonucleotide **4**), which comprised 45% (Fig. 8A,C). After 4 h, efficient cleavage of miR-17 went up to 47%, as observed at the sites C_17_**-**A_18_ and U_21_**-**A_22_. After 8 h, miRNA was already destroyed by 95%, mainly at cleavage sites of conjugate **4** (Fig. 8).

Thus, designed conjugates did not affect the functioning of RNase H, while the presence of RNase H in the reaction mixture increased the catalytic activity of the conjugate and provided an effective cleavage of miRNA in a 2-fold excess. In other words, the synergetic action of conjugate **4** and RNase H was observed when the activity of the conjugate increased 11-fold, while the activity of RNase H remained almost unaltered. It can be assumed that low ribonuclease activity of conjugate **4** can be associated with the formation of a number of unproductive complexes between the catalytic domain and adjacent RNA, including those with a double-stranded part of the complex due to an increased electrostatic field. When RNase H is added, it interacts with the central part of the DNA/RNA duplex, and probably displaces the catalytic domain and peptide, so the peptide forced to interact more often with the single-stranded 3′-region of RNA, and cleaved it. Moreover, the cleavage of miRNA by RNase H can apparently facilitate dissociation of miRNA fragments from the complex with the conjugate and the attack of the next molecule, promoting the true catalytic mode of action of the conjugate.

The obtained data indicated that within the cell miRNA degradation occurs presumably due to both the ribonuclease activity of a specific miRNase and by recruiting RNase H. The oligonucleotide part of the conjugate efficiently binds with miRNA, providing cleavage of the 5′-end of the miRNA molecule by RNase H, and the 3′-end of the molecule by the catalytic construction of the conjugate. The use of the antisense oligonucleotide shortened to 14 nt apparently shifts the main RNase H cleavage sites from the center to the 5′-end, in particular to the seed region (bases 2–8), which is known to be the a “canonical” determinant in miRNA function^33,34^. In addition, an important role of the 3′-end of miRNA in bases 13–18 was reported, elucidating that this region represents a “3′-compensatory” or “beneficial 3′-paring” site in miRNA-mRNA target recognition and is also crucial for miRNA specificity and functioning^34–37^.

## Discussion

The objectives behind the work were (i) development of biodegradable molecules, (ii) the high stability of the conjugates based on its constituent natural structural components, (iii) multiple reaction turnover, and (iv) in addition to own ribonuclease activity of the conjugate, involvement of intracellular enhancers RNase H, RNase L and/or steric blockage all together. As a result we developed novel miRNases — peptide-oligonucleotide conjugates targeted to highly oncogenic miR-21 and miR-17. miRNases consist of high stable hairpin oligonucleotides comprising the sequence complementary to the particular miRNA and small chemical ribonuclease NH_2_**-**Gly(ArgLeu)_4_-TCAA conjugated via flexible linkers on the base of di- or tetraethylene glycol residues.

When designing conjugates, we used the double principle of protecting their structure from nuclease degradation and increasing the efficiency of its functioning as artificial ribonuclease. As non-toxic and biodegradable component of antisense oligonucleotide in the conjugates capable to increase stability towards nucleases we use a DNA fragment GCGAAAGC, which was shown to adopt stable mini-hairpin structure and display extraordinary properties: high thermostability^21^ and robustness against nuclease digestion^22^. Taking into account these properties we decide to introduce this structure at the 3′-end of oligonucleotide in the conjugates to protect from nuclease degradation. The 5′-end of the oligonucleotide was protected by catalytic construction. We did not introduce any modifications into the structure of the oligonucleotide that could increase the stability of the duplex and thus prevent the dissociation of the oligonucleotide after the cleavage event, and, therefore, could affect reaction turnover.

We can assert that conjugates designed in our study cleave RNA in a non-random manner. Our data revealed that conjugation of small ribonuclease to oligonucleotide did not decrease its ribonuclease activity: miRNases cleaved non-complementary fragment of RNA HIV-1 with cleavage efficiency comparable to that of small chemical ribonuclease and displayed the same Pyr-A specificity of cleavage. In the case of complementary RNA substrates conjugates are forced to cleave all sensitive linkages located nearby catalytic domain of the conjugate. These are C-U, U-G and U-U linkages (united under a common name Pyr-X linkages) which it is not unexpected because sensitivity of these motifs to cleavage was shown to decrease in range U-A = C-A»Pyr-C > Pyr-G > Pyr-U^31^.

Conjugates of similar peptide and short non-targeted oligonucleotides of random sequence are capable of cleaving non-complementary RNAs, as it was shown in a number of studies^17,18,38^. Nevertheless, when oligonucleotide within the conjugate structure can form complementary complex with RNA target RNA molecule is cleaved predominantly at the targeted site^11,16^.

Analysis of the structure - activity relationship showed that the structure of oligonucleotide in the conjugates determined cleavage efficiency, as well as the pattern of miRNA cleavage. So, shortening of the oligonucleotide part complementary to the miRNA target from 14 to 12 nucleotides resulted in increased efficiency of site-specific cleavage by the conjugates. The length of the stem in oligonucleotide did not affect the specificity of miRNA cleavage by the conjugates but is important for cleavage efficiency. With the example of conjugate **2** and conjugate **3**, it can be seen that lengthening of the stem from 6 to 9 base pairs significantly decreased the efficiency of target miRNA cleavage. Thus, it is clear that a strong binding of the conjugate with miRNA leads to a drop in its activity, highly likely due to its slow dissociation from the complex or rigid fixation of the catalytic construction. In the same time in more breathing and flexible complex like in the case of conjugate **2**, the catalytic construction turns out to be structurally and thermodynamically in a more favorable position for the implementation of the cleavage. The absence of a hairpin in the oligonucleotide part of the conjugate resulted in alteration of cleavage specificity: cleavage occurs not in the 3′-region of miR-21 whereinto the conjugate catalytic domain was targeted, but at the first phosphodiester bond from the 5′-region of a molecule, as shown for conjugate **1**. This resulted from the breathing structure of the complex formed between miRNA and linear antisense oligonucleotide of 14 nts in length, and the possibility of the catalytic domain to reach the 5′-region of miRNA.

It was found that the length of the linker group is also important for cleavage activity of the conjugates. So, shortening of the linker group from two to one diethylene glycol residues leads to a significant decrease in ribonuclease activity of the conjugate. Our data showed that the ribonuclease activity of the conjugate is strongly determined by the presence of a short oligonucleotide TCAA in its structure: removing the oligonucleotide TCAA from the catalytic construction entirely inhibited its catalytic activity.

One of the important finding of this study is the fact that peptide-oligonucleotide conjugates efficiently silence miRNA target, acting synergistically with RNase H. In cell culture, conjugate **4** targeted to miR-17 caused 5-fold retardation of cell proliferation, efficiently reduced miR-17 level to 20% of control, showed a 2-fold advantage in inhibition efficiency in comparison with antisense oligonucleotide **4**, and resulted in a 2.2-fold increase of the level of miR-17 protein target E2F1.

This data correlates well with the observed significant enhancement of the miRNA cleavage rate by the conjugate in the presence of RNase H. Recruitment of RNase H by the conjugate leads to an 11-fold increase of miR-17 cleavage at the conjugate sites *in vitro* and significantly more effective inhibition of particular miRNA within the cells. The pronounced silencing effect observed for conjugate **4** compared to antisense oligonucleotide **4**, could also depend on the increased biostability of conjugate **4** with respect to corresponding oligonucleotide.

### Conclusions and perspectives

General rules for engineering of the best miRNases can include optimal balance between stability of their structural components, sufficient binding efficiency of miRNase with miRNA target and quick dissociation of miRNase from complex after catalytic act that provide multiple reaction turnover, selectivity of miRNases toward particular miRNA target, low toxicity, absence of off-target effects. The structural features of our best miRNase can be summarized as follows: miRNase include an antisense oligonucleotide to ensure selectivity of binding to the miRNA target, hairpin structure at the 3′-end of the molecule providing high resistance of miRNases to nucleases and a catalytic fragment at the 5′-end attached via two diethylene glycol residues. Our miRNases consists of biodegradable molecules that allow evading problems with toxicity. Moreover, designed miRNases recruit intracellular RNase H providing multifold enhancement of cleavage efficiency of RNA target.

The obtained data allows us to consider the developed miRNases as promising inhibitors of oncogenic miRNAs in cell cultures, which in the future can be used for the reversion of malignant phenotypes of tumors.

## Materials and Methods

### Synthesis of the conjugates

Oligodeoxyribonucleotides for conjugation with peptide were designed according to Patutina *et al*.^20^ and synthesized by the standard phosphoramidite protocol on an ASM-800 synthesizer (Biosset, Russia) using solid support, nucleoside phosphoramidites, and chemical phosphorylation reagent from Glenn Research (USA). Normucleotidic fragments (5′-phosphate group, olygoethylene glycol linkers) were introduced into the molecules with commercial [3-(4,4′-dimethoxytrityloxy)−2,2-dicarboxyethyl]propyl-(2-cyanoethyl)-(N,N-diisopropyl)-phosphoramidite and corresponding 9-O-dimethoxytrityl-ethylene glycol,1-[(2-cyanoethyl)-(N,N-diisopropyl)]-phosphoramidites during oligonucleotide synthesis using the standard synthetic method recommended by the synthesizer manufacturer. Oligonucleotides were isolated by RP-HPLC (ZORBAX Eclipse XDB-C18 column, Agilent, USA) according to standard protocols.

The conjugates were synthesized via a phosphamide bond formation between the 5′-terminal phosphate of oligonucleotide and the N-terminal α-amino group of the oligopeptide [LeuArg]_4_-Gly-NH_2_ (Almabion Ltd, Russia), as described by Mironova *et al*.^17^ and, Zarytova *et al*.^39^. The conjugates were isolated by RP-HPLC on the same columns. The homogeneity of the conjugates was tested by analytical electrophoresis in 20% denaturing polyacrylamide gel containing 8 M urea, followed by visualization with Stains-all dye. The homogeneity of the oligonucleotides and the conjugates was 95–98%. The identity and purity of all peptide-oligonucleotide conjugates were confirmed by denaturing electrophoresis, RP-HPLC, MALDI and ESI-MS (Table 1). Conjugates were identified in free form, and measured masses have shown good agreement with theoretical values.

### 5′-RNA labeling

miR-21 5′-UAG CUU AUC AGA CUG AUG UUG A-3′, miR-21^hybrid^ 5′-UAG CUU AUC AGA CUC AUC AUG A-3′, and miR-17 5′-CAA AGU GCU UAC AGU GCA GGU AG-3′ were synthesized by Dr. Meschaninova (ICBFM SB RAS). RNA HIV-1 was prepared by *in vitro* transcription with T7 RNA polymerase (Thermo Scientific, USA) as described in Mironova *et al*.^17^. HIV-1 RNA transcript was dephosphorylated using bacterial alkaline phosphatase BAP (Thermo Scientific, USA) according to a described protocol^38^. 5′-End labeling using [γ^32^P]ATP (Biosan Co, Russia) and T4 polynucleotide kinase (Thermo Scientific, USA) and isolation of 5′-[^32^P]-RNA were carried out according to the procedure described by Silberklang *et al*^40^. and Mironova *et al*.^38^.

### Nuclease resistance studies

Oligonucleotide **1**, oligonucleotide **3** or conjugate **3** in concentration 0.1 µg/µl were incubated in Dulbecco’s Modified Eagle Medium (DMEM) containing 10% fetal bovine serum (FBS) at 37 °C for 48 h. Aliquots (10 µl) were taken at 0, 2, 4, 8, 24 and 48 h and reaction was quenched by adding 10 µl of 8 M urea and immediate freezing in liquid nitrogen. Samples were defrosted and analyzed in 12% PAAG/8 M urea using TBE as running buffer. Then electrophoresis gels were stained with Stains-All (MP Biomedicals, USA) and photographed using gel documentation system (Vilber Lourmat, France).

### Gel-retardation assay

The reaction mixture (5 µl) containing 50 mM Tris-HCl, pH 7.0, 0.2 M KCl, 1 mM EDTA, 10^5^ cpm (Cherenkov’s counting) of [^32^P]-miR-21, 1 μM unlabeled miR-21, and antisense oligonucleotide (asON) or conjugate at a concentration ranging from 0.5 to 10 μM, was incubated at 37 °C for 1 h and quenched by adding an equal volume of the loading buffer (20% ficoll, 0.025% bromophenol blue, and 0.025% xylene cyanol). The samples were loaded onto the running gel immediately after quenching the reaction with an interval of 1 min. Formation of the complex miR-21/asON or miR-21/conjugate was analyzed by electrophoresis in 15% native PAAG at 4 °C. To obtain quantitative data, gels were dried and analyzed using Molecular Imager FX (Bio-Rad, USA). The extent of binding of oligonucleotide to miR-21 was determined using Quantity One software as a ratio of radioactivity measured in the complex to the total radioactivity applied onto the gel lane.

### RNA cleavage assay

The reaction mixture (5 µl) contained 10^5^ cpm (Cherenkov’s counting) of [^32^P]-RNA, 1 μM unlabeled RNA, and one of the conjugates at a concentration ranging from 1 to 100 μM, 50 mM Tris-HCl, pH 7.0, 0.2 M KCl, and 1 mM EDTA. The mixture was incubated at 37 °C (for various times) and quenched by precipitation of RNA with 2% LiClO_4_ in acetone (50 µl). RNA was collected by centrifugation and dissolved in loading buffer (8 M urea, 0.025% bromophenol blue, and 0.025% xylene cyanol). RNA cleavage products were analyzed in 18% PAAG/8 M urea using TBE (100 mM Tris-borate, pH 8.3, 2 mM EDTA) as the running buffer. To identify cleavage sites, imidazole and T1-ladders produced by partial RNA cleavage with 2 M imidazole buffer (pH 7.0)^41^ and with RNase T1^42^, respectively, were run in parallel. To obtain quantitative data, gels were dried and analyzed using Molecular Imager FX (Bio-Rad, USA). The total extent of RNA cleavage and the extent of RNA cleavage at each individual site were determined with Quantity One software.

### Cleavage of duplexes miR-17/ON and miR-17/conjugate by RNase H

The reaction mixture (10 µl) contained 10^5^ cpm (Cherenkov’s counting) of [^32^P]-miR-17, 10 µM unlabeled miR-17, oligonucleotide **4** or conjugate **4** at a concentration of 5 µM, 50 mM Tris-HCl, pH 7.0, 0.2 M KCl, and 1 mM EDTA was incubated at 37 °C for 20 min. Then, RNase H (Thermo Scientific, USA) was added to the reaction mixture to a final concentration 100 U/ml and incubated at 37 °C. At different time points, 30 min and 1, 2, 4, 8, and 24 h, an aliquot was taken and the reaction was quenched by precipitation of RNA with 2% LiClO_4_ in acetone. RNA cleavage products were analyzed by electrophoresis in denaturing gel as described above.

### Transfection of tumor cells with conjugates

Murine B16 melanoma cells (1.5–1 × 10^5^ per well of 24-well plate) were pre-seeded in DMEM containing 10% FBS a day before transfection and were incubated at 37 °C in a humidified atmosphere with 5% CO_2_. Before the transfection, the medium was replaced by serum-free and antibiotic-free DMEM, and cells were incubated for 4 h with 0.05–1 µM of conjugate **4** precomplexed with Lipofectamine 2000 (Invitrogen, USA) in Opti-MEM medium according to manufacturer’s instructions. Then, the medium was replaced by culture medium containing 10% FBS and 1% antibiotic antimycotic solution (10000 mg/mL streptomycin, 10000 IU/mL penicillin, and 25 µg/mL amphotericin), and the cells were cultivated for 24 h under the same conditions. After 24 h, total RNA was isolated from the cells using TRIzol Reagent (Invitrogen, USA) according to the manufacturer’s protocol.

### qPCR

Expression of miR-17, miR-21, miR-18a and let-7g in B16 cells was analyzed using stem-loop PCR technology^43,44^. cDNA synthesis was carried out using SuperScript III reverse transcriptase (SSIII RT, Invitrogen, USA) as previously described^45^. The RT and PCR primers used in the study are presented in Table S1 (Supplementary information). PCR amplification was carried out in a total volume of 20 µl, using Maxima Hot Start Taq DNA polymerase (Thermo Scientific, USA), 1 × PCR Buffer, 1.5 mM MgCl_2_, 0.2 mM dNTPs, 1 × EvaGreen (Biotium, Hayward, USA), and 0.2 mM of PCR sense and antisense primers. The reaction was performed with initial preheating at 94 °C for 4 min and 40 cycles of 94 °C for 40 s, 61 °C for 30 s, and 72 °C for 30 s, followed by melting point determination. The obtained PCR data were analyzed using standard Bio-Rad iQ5 v.2.0 software. For each sample, the threshold cycle (Ct) was determined. Quantitative assessment of the level of transcripts representation and relative miRNA expression was performed by comparing the Ct values for miR-17 and a reference U6.

### xCELLigence real-time analysis of cell proliferation

Proliferation experiments were performed using an xCELLigence real-time cell analysis (RTCA) system (ACEABiosciences, USA) in an atmosphere of 5% CO_2_ at 37 °C. B16 melanoma cells were seeded at a concentration of 5 × 10^3^ cells per well of 16-well E-Plates. The following day, the cells were transfected with conjugate **4**, control conjugate **7** or oligonucleotide **4** at 1 µM concentration using Lipofectamine™ 2000 (Invitrogen, USA). In 4 h after transfection, the medium in the wells was replaced with DMEM medium containing 10% of FBS and 1% of antibiotics antimycotic solution. Cell proliferation experiments were run for 120 h and Cell Index was monitored every 30 min for the whole experiment duration. Four replications of each cell densities were used in the cell proliferation experiment.

### Western blot

Cell lysates were separated in 12.5% SDS-PAGE and transferred to a PVDF membrane using a semi-dry transfer. The membranes were blocked for 1 h in 3% nonfat dried milk in TBST (20 mM Tris- HCl, pH 7.6,137mMNaCl, 0.1% Tween), and incubated with primary antibodies against E2F1 (ab179445, Abcam, UK, 1:500) and reference protein GAPDH (ab9485, Abcam, UK, 1:1000) at +4 °C for night. After three washes with TBST, membranes were incubated with secondary HRP-conjugated goat anti-rabbit antibodies (ab6721, Abcam, UK) at room temperature for 1 h. After three washes with TBST luminescence detection was performed by Versadoc 4000 MP (Bio-Rad, USA) using Chemiluminescent reagent kit (Abcam, USA). Data were analyzed using GelPro 4.0 software (Media Cybernetics, USA).

### Statistics

The data obtained were statistically processed using the Student’s t-test (two-tailed, unpaired) and one-way ANOVA. Post-hoc testing was completed using post-hoc Tukey test; p < 0.05 was considered to be statistically significant. The statistical package STATISTICA version 10.0 was used for analysis.

## Electronic supplementary material

Supplementary information

## Data Availability

The authors declare all data availability.

## References

[1] Hanahan D, Weinberg RA (2011). Hallmarks of cancer: the next generation. Cell.

[2] Grijalvo S, Alagia A, Jorge AF, Eritja R (2018). Covalent strategies for targeting messenger and non-coding RNAs: an updated review on siRNA, miRNA and antimiR conjugates. Genes (Basel).

[3] Ambros V (2004). The functions of animal microRNAs. Nature.

[4] Bartel DP (2004). MicroRNAs: genomics, biogenesis, mechanism, and function. Cell.

[5] Pillai RS (2005). Inhibition of translational initiation by Let-7 microRNA in human cells. Science.

[6] Dalmay T, Edwards DR (2006). MicroRNAs and the hallmarks of cancer. Oncogene.

[7] Esquela-Kerscher A, Slack FJ (2006). Oncomirs – microRNAs with a role in cancer. Nat. Rev. Cancer.

[8] Gambari R, Brognara E, Spandidos DA, Fabbri E (2016). Targeting oncomiRNAs and mimicking tumor suppressor miRNAs: Νew trends in the development of miRNA therapeutic strategies in oncology. Int. J. Oncol..

[9] Nguyen DD, Chang S (2018). Development of novel therapeutic agents by inhibition of oncogenic microRNAs. Int. J. Mol. Sci..

[10] Gaglione M (2011). PNA-based artificial nucleases as antisense and anti-miRNA oligonucleotide agents. Mol. Biosyst..

[11] Patutina OA (2017). miRNases: novel peptide-oligonucleotide bioconjugates that silence miR-21 in lymphosarcoma cells. Biomaterials.

[12] Danneberg F (2015). Sequence-specific RNA cleavage by PNA conjugates of the metal-free artificial ribonuclease tris(2-aminobenzimidazole). Beilstein J. Org. Chem..

[13] Kuznetsova IL, Zenkova MA, Gross HJ, Vlassov VV (2005). Enhanced RNA cleavage within bulge-loops by an artificial ribonuclease. Nucleic Acids Res..

[14] Patino N (2002). Modelling, synthesis and biological evaluation of an ethidium-arginine conjugate linked to a ribonuclease mimic directed against TAR RNA of HIV-1. Eur. J. Med. Chem..

[15] Baker BF (1999). Oligonucleotide-europium complex conjugate designed to cleave the 2′ cap structure of the ICAM-1 transcript potentiates antisense activity in cells. Nucleic Acids Res..

[16] Williams A (2015). Peptidyl-oligonucleotide conjugates demonstrate efficient cleavage of RNA in a sequence-specific manner. Bioconjug. Chem..

[17] Mironova NL (2004). Covalently attached oligodeoxyribonucleotides induce RNase activity of a short peptide and modulate its base specificity. Nucleic Acids Res..

[18] Mironova NL (2006). G-specific RNA-cleaving conjugates of short peptides and oligodeoxyribonucleotides. J. Biomol. Struct. Dyn..

[19] Sunami T (2004). Structure of d(GCGAAAGC) (hexagonal form): a base-intercalated duplex as a stable structure. Acta Crystallogr. D. Biol. Crystallogr..

[20] Patutina OA, Miroshnichenko SK, Lomzov AA, Mironova NL, Zenkova MA (2017). Search for oligonucleotides selectively binding oncogenic miR-21. Rus. J. Bioorgan. Chem..

[21] Hirao I, Nishimural Y, Tagawa Y, Watanabe K, Miura K (1992). Extraordinarily stable mini-hairpins: electrophoretical and thermal properties of the various sequence variants of d(GCGAAAGC) and their effect on DNA sequencing. Nucleic Acids Res..

[22] Hirao I (1989). Extraordinary stable structure of short single-stranded DNA fragments containing a specific base sequence: d(GCGAAAGC). Nucleic Acids Res..

[23] Tang JY, Temsamani J, Agrawal S (1993). Self-stabilized antisense oligodeoxynucleotide phosphorothioates: properties and anti-HIV activity. Nucleic Acids Res..

[24] Vermeulen A (2007). Double-stranded regions are essential design components of potent inhibitors of RISC function. RNA.

[25] Lennox KA, Behlke MA (2010). A direct comparison of anti-microRNA oligonucleotide potency. Pharm. Res..

[26] Minami Y (2014). SS18-SSX-regulated miR-17 promotes tumor growth of synovial sarcoma by inhibiting p21WAF1⁄CIP1. Cancer Sci..

[27] Kumarswamy R, Volkmann I, Thum T (2011). Regulation and function of miRNA-21 in health and disease. RNA Biol..

[28] Buscaglia LE, Li Y (2011). Apoptosis and the target genes of microRNA-21. Chin. J. Cancer..

[29] Mironova NL (2002). Sequence-specific cleavage of the target RNA with oligonucleotide-peptide conjugates. Russ. Chem. Bull..

[30] Mukaiyama T, Matsueda R, Suzuki M (1970). Peptide synthesis via the oxidation-reduction condensation by the use of 2,2*′*-dipyridyldisulfide as an oxidant. Tetrahedron Lett..

[31] Kierzek R (1992). Hydrolysis of oligoribonucleotides: influence of sequence and length. Nucleic Acids Res..

[32] Meng P, Ghosh R (2014). Transcription addiction: Can we garner the Yin and Yang functions of E2F1 for cancer therapy. Citation: Cell Death and Disease.

[33] Lai EC (2002). Micro RNAs are complementary to 3′ UTR sequence motifs that mediate negative post-transcriptional regulation. Nat. Genet..

[34] Brennecke J, Stark A, Russell RB, Cohen SM (2005). Principles of microRNA-target recognition. PLoS Biol..

[35] Grimson A (2007). MicroRNA targeting specificity in mammals: determinants beyond seed pairing. Mol. Cell..

[36] Robertson B (2010). Specificity and functionality of microRNA inhibitors. Silence.

[37] Wang Y (2009). Nucleation, propagation and cleavage of target RNAs in Ago silencing complexes. Nature.

[38] Mironova NL (2007). RNase T1 mimicking artificial ribonuclease. Nucleic Acids Res..

[39] Zarytova VF, Ivanova EM, Yarmoluk SN, Alekseeva IV (1988). Synthesis of oligonucleotidyl-(5′-N)-peptides containing arginine. Biopolym. Cell.

[40] Silberklang F, Gillum AM, RajBhandary UL (1979). Use of *in vitro* 32P-labeling in the sequence analysis of nonradioactive tRNAs. Methods Enzymol..

[41] Vlasov AV, Vlasov VV, Giege R (1996). RNA hydrolysis catalyzed by imidazole as a reaction for studying the secondary structure of RNA and complexes of RNA with oligonucleotides. Dokl. Akad. Nauk..

[42] Donis-Keller H, Maxam AM, Gilbert W (1977). Mapping adenines, guanines and pyrimidines in RNA. Nucleic Acids Res..

[43] Chen C (2005). Realtime quantification of microRNAs by stem-loop RT-PCR. Nucleic Acids Res..

[44] Varkonyi-Gasic E, Wu R, Wood M, Walton EF, Hellens RP (2007). Protocol: a highly sensitive RT-PCR method for detection and quantification of micro-RNAs. Plant Methods.

[45] Mironova N (2013). MicroRNA drop in the bloodstream and microRNA boost in the tumour caused by treatment with ribonuclease A leads to an attenuation of tumour malignancy. PLoS One.

