# Supplementary material for: Peptide-oligonucleotide conjugates exhibiting pyrimidine-X cleavage specificity efficiently silence miRNA target acting synergistically with RNase H
Source: Sci Rep. 2018 Oct 9;8:14990. doi: 10.1038/s41598-018-33331-z (PMC6177439; doi:10.1038/s41598-018-33331-z)
Supplement: Supplementary file 1 — Supplementary information [file 41598_2018_33331_MOESM1_ESM.docx]

**Peptide-oligonucleotide conjugates exhibiting pyrimidine-X cleavage specificity efficiently silence miRNA target acting synergistically with RNase H**

Patutina O.A., Bazhenov M.A., Miroshnichenko S.K., Mironova N.L., Pyshnyi D.V., Vlassov V.V., and Zenkova M.A.*

*Institute of Chemical Biology and Fundamental Medicine SB RAS, Lavrentiev ave., 8, Novosibirsk, 630090, Russia*

*Corresponding author: Zenkova M.A.; e-mail: [marzen@niboch.nsc.ru](mailto:marzen@niboch.nsc.ru); phone: +7(383) 3635160; fax: +7(383) 363-51-53.


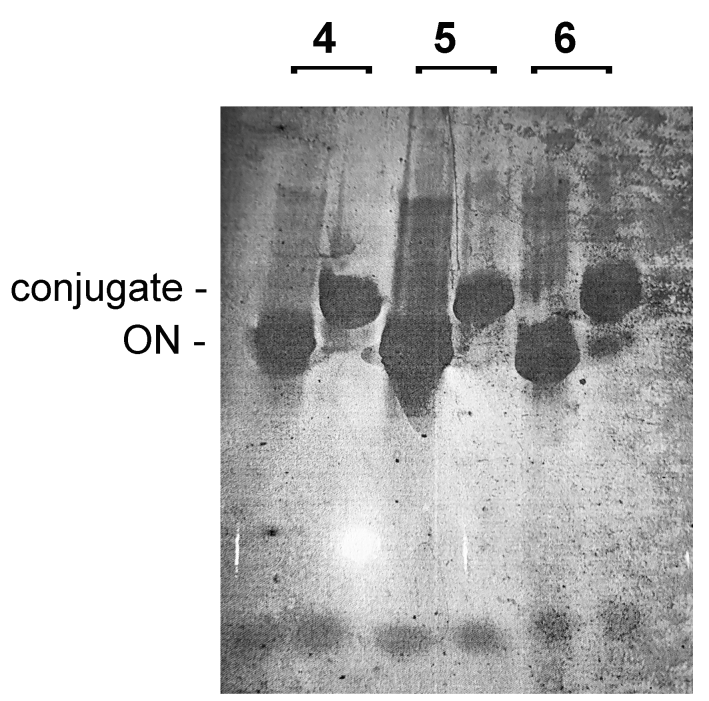


**Figure S1.** Typical 20% polyacrylamide/8 M urea gel colored by Stains-all dye showing the homogeneity of conjugates **4**, **5** and **6** and the changes in conjugates mobility in comparison with corresponding oligonucleotides.


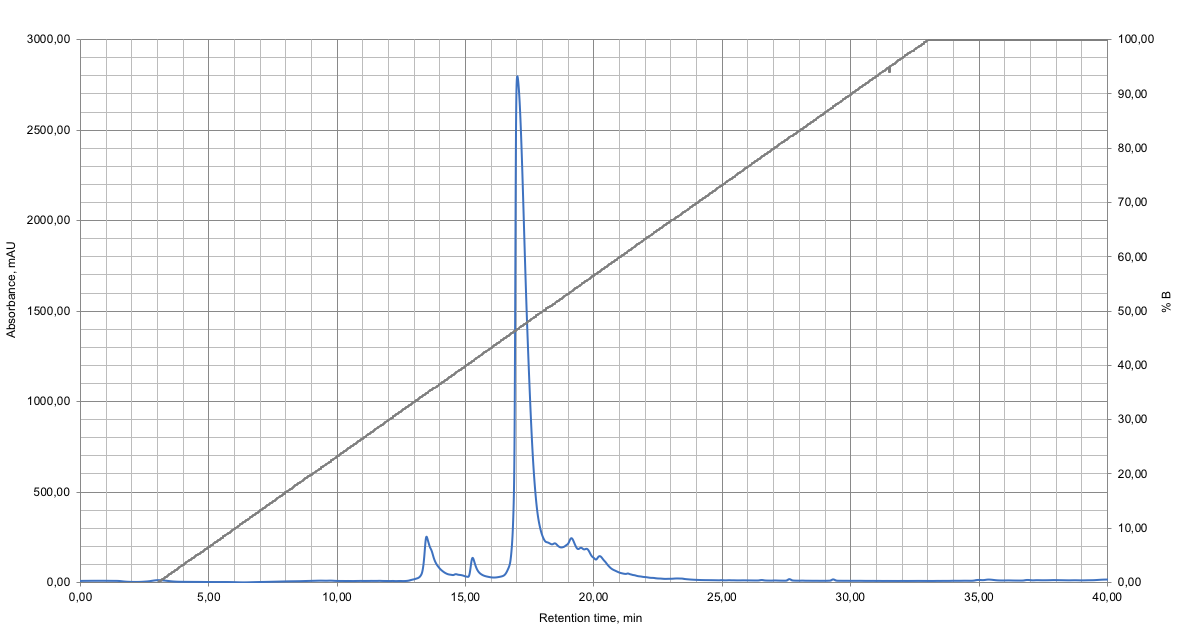


**Figure S2.** Typical HPLC profile (at 260 nm) of peptide-oligonucleotide conjugates with the example of NH_2_—Gly(ArgLeu)_4_—P—TCAA—(DEG)_2_—**CTG TAA GCA CTT TG**G TCA GCG AAA GCT GAC -3’ (conjugate **4**).


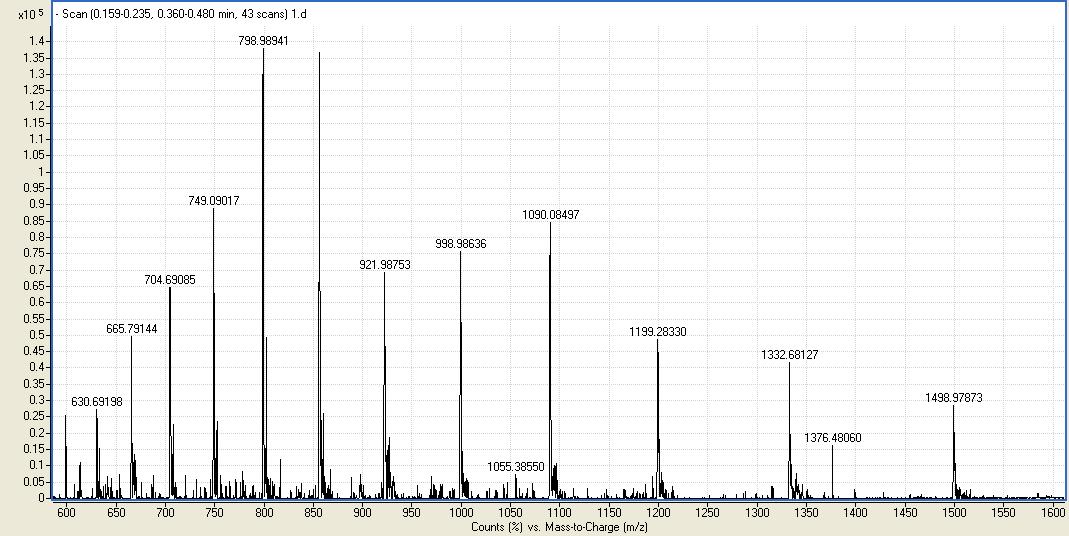


**Figure S3.** Typical ESI spectrum (negative mode) for peptide-oligonucleotide conjugates with the example of NH_2_—Gly(ArgLeu)_4_—P—TCAA—(DEG)_2_—**CTG TAA GCA CTT TG**G TCA GCG AAA GCT GAC -3’ (conjugate **4**).


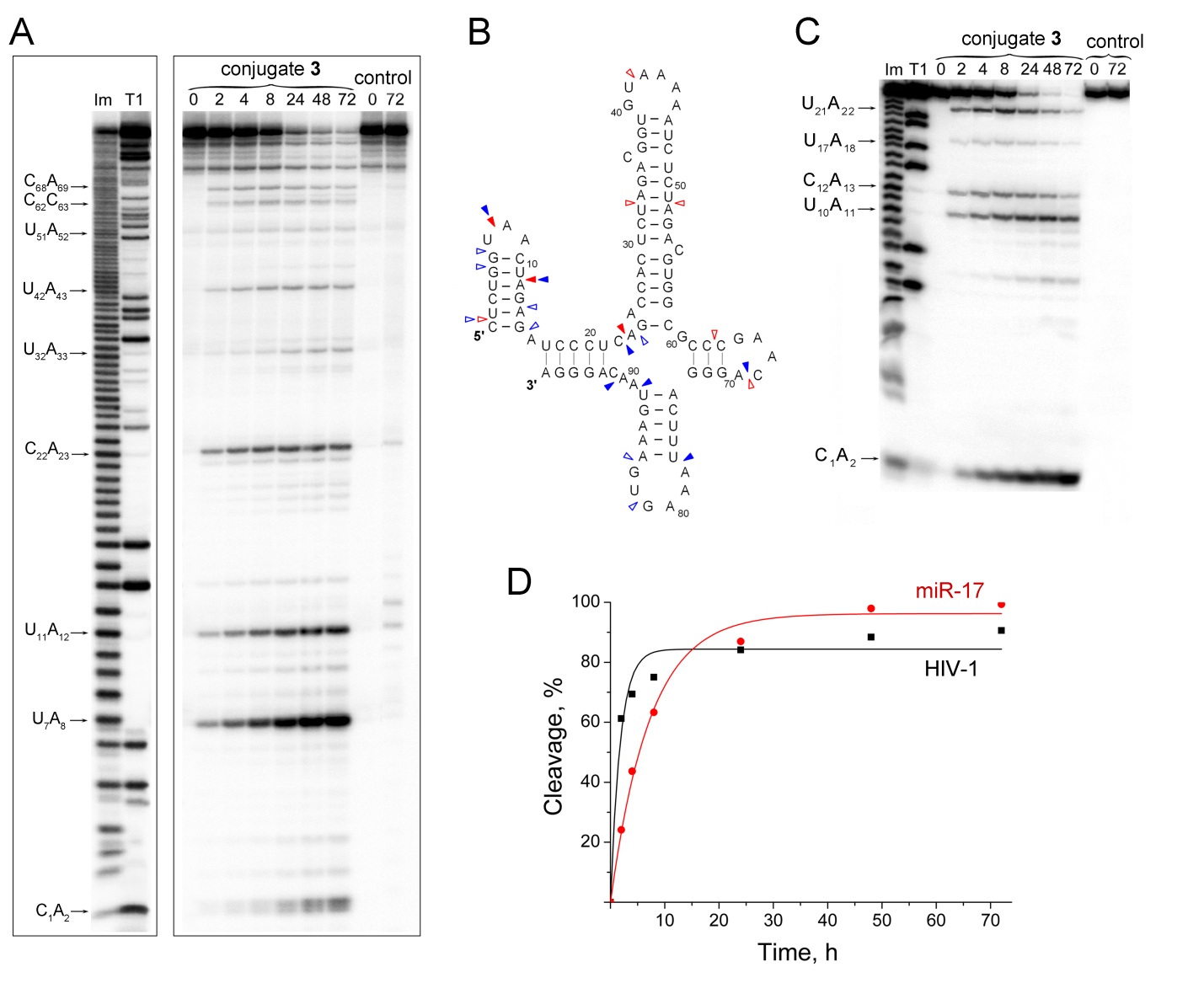


**Figure S4.** Cleavage of non-complementary RNA targets by conjugate **3**. **A.** Cleavage of 5’-[^32^P]-labeled 96 nts fragment of HIV-1 RNA by conjugate **3**. Autoradiograph of 15% polyacrylamide/8 M urea gel, showing the pattern of HIV-1 RNA cleavage by the conjugate. Lanes Im and T1 — imidazole ladder and partial RNA digestion with RNase T1, respectively; control — RNA incubated in the absence of conjugates for 0 and 72 h, respectively. HIV-1 RNA (1 µM) and conjugate **3** (30 µM) were incubated at 37°C for 0–72 h. Incubation times are shown at the top. The images enclosed by lines are parts of the different gels. **B.** Secondary structure of the fragment of HIV-1 RNA. Red triangles indicate sites of cleavage by the conjugate **3**, blue triangles indicate sites of cleavage by small ribonuclease NH_2_-Gly(ArgLeu)_4_-TCAA (according to ^16^). Strong and weak cleavage sites are shown as full and open symbols, respectively. **C.** Cleavage of 5’-[^32^P]-miR-17 by conjugate **3**. Autoradiograph of 18% polyacrylamide/8 M urea gel, showing the pattern of miR-17 cleavage by the conjugate. miR-17 (1 µM) and conjugate **3** (30 µM) were incubated at 37°C for 0–72 h. **D.** Kinetics of HIV-1 RNA and miR-17 cleavage by conjugate **3**.


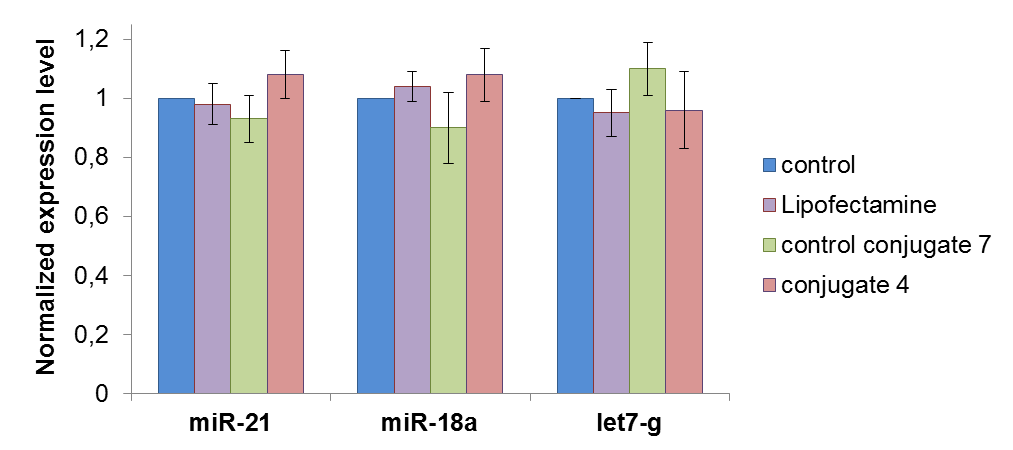


**Fig. S5.** qPCR analysis of the expression levels of miR-21, miR-18a and let7-g in melanoma B16 cells 24 after transfection with 1 µM of conjugate **4** and control conjugate **7**. Transfection was performed using Lipofectamine2000. The expression of miRNAs was normalized to *U6*. Data are given as mean calculated from three independent experiments ± SEM.

**Table S1.** RT and PCR primers used in the study.

| **Name** | **Oligodeoxyribonucleotide sequence (5’→3’)** |
| --- | --- |
| RT-miR-17 | GTCGTATCCAGTGCAGGGTCCGAGGTATTCGCACTGGATACGACCTACCTGCAC |
| RT-let-7g RT | GTCGTATCCAGTGCAGGGTCCGAGGTATTCGCACTGGATACGACAACTGTACAA |
| RT-miR-21 | GTCGTATCCAGTGCAGGGTCCGAGGTATTCGCACTGGATACGACTCAACATCAG |
| RT-miR-18a | GTCGTATCCAGTGCAGGGTCCGAGGTATTCGCACTGGATACGACTATCTGCACT |
| RT-U6 | GTCGTATCCAGTGCAGGGTCCGAGGTATTCGCACTGGATACGACAAAAATATGGAACG |
| miR-17 sense | AGACAAAGTGCTTACAGTGC |
| let-7g sense | AACGCTGAGGTAGTAGTTTGT |
| miR-21 sense | AGACTAGCTTATCAGACTGA |
| miR-18a sense | GCGTAAGGTGCATCTAGTG |
| U6 sense | CTCGCTTCGGCAGCACA |
| Universal antisense | GTGCAGGGTCCGAGGT |
